# FGF/FGFR signaling in health and disease

**DOI:** 10.1038/s41392-020-00222-7

**Published:** 2020-09-02

**Authors:** Yangli Xie, Nan Su, Jing Yang, Qiaoyan Tan, Shuo Huang, Min Jin, Zhenhong Ni, Bin Zhang, Dali Zhang, Fengtao Luo, Hangang Chen, Xianding Sun, Jian Q. Feng, Huabing Qi, Lin Chen

**Affiliations:** 1 Desartment of Wound Repair and Rehabilitation Medicine, State Key Laboratory of Trauma, Burns and Combined Injury, Trauma Center, Research Institute of Surgery, Daping Hospital, Army Medical University, Chongqing, China; 2 Department of Biomedical Sciences, Texas A&M University College of Dentistry, Dallas, TX 75246 USA

**Keywords:** Diseases, Developmental biology

## Abstract

Growing evidences suggest that the fibroblast growth factor/FGF receptor (FGF/FGFR) signaling has crucial roles in a multitude of processes during embryonic development and adult homeostasis by regulating cellular lineage commitment, differentiation, proliferation, and apoptosis of various types of cells. In this review, we provide a comprehensive overview of the current understanding of FGF signaling and its roles in organ development, injury repair, and the pathophysiology of spectrum of diseases, which is a consequence of FGF signaling dysregulation, including cancers and chronic kidney disease (CKD). In this context, the agonists and antagonists for FGF-FGFRs might have therapeutic benefits in multiple systems.

## Introduction of the FGF/FGFR signaling

**Fig. 1:**
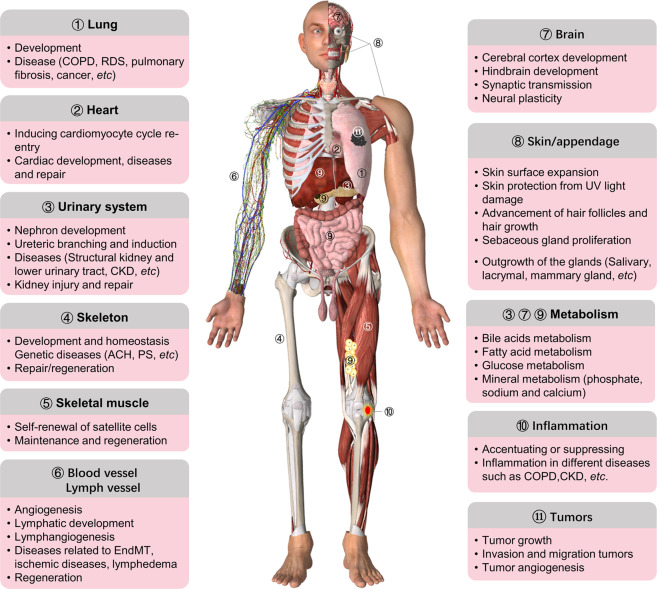
Summary of the main roles of FGF/FGFR signaling in organ development, metabolism, and disease. FGF/FGFR signaling participates in the development of almost all organ such as lung, heart, urinary system, brain, skeleton, muscle, and skin/appendage, as well as angiogenesis and lymphangiogenesis. FGFs/FGFRs also have important effects on tissue repair, regeneration, and inflammation. Furthermore, endocrine FGFs play critical roles in metabolism by regulating kidney, liver, brain, intestine, and adipose tissue. The malfunctions of FGF/FGFR signaling lead to multiple kinds of diseases, such as genetic diseases, cancer, COPD, and CKD. The roles of FGF signaling in appendage development, such as epidermis, hair, and glands, and so on, is not mentioned in this review. ACH achondroplasia, CKD chronic kidney disease, COPD chronic obstructive pulmonary disease, PS Pfeiffer syndrome, RDS respiratory distress syndrome, EndMT endothelial-to-mesenchymal transition

Fibroblast growth factors (FGFs) are broad-spectrum mitogens and regulate a wide range of cellular functions, including migration, proliferation, differentiation, and survival. It is well documented that FGF signaling plays essential roles in development, metabolism, and tissue homeostasis. The malfunction of FGF/FGF receptor (FGFR) signaling axis is observed in a variety of human diseases, such as congenital craniosynostosis and dwarfism syndromes, as well as chronic kidney disease (CKD), obesity, insulin resistance, and various tumors (Fig. 1).

FGF family is one of the most diverse growth factor groups in vertebrates. In mice and humans, 22 FGF ligands have been identified. Based on sequence homology and phylogeny, the 18 canonical mammalian FGFs are divided into six subfamilies, including five paracrine subfamilies and one endocrine subfamily.^1^ Five paracrine subfamilies contain the FGF1 subfamily (FGF1 and FGF2), the FGF4 subfamily (FGF4, FGF5, and FGF6), the FGF7 subfamily (FGF3, FGF7, FGF10, and FGF22), the FGF8 subfamily (FGF8, FGF17, and FGF18), and the FGF9 subfamily (FGF9, FGF16, and FGF20). The FGF19 subfamily (FGF19, FGF21, and FGF23) signals in an endocrine manner.^1^

FGFs exert their pleiotropic effects by binding and activating high-affinity tyrosine kinase receptors that are coded by four genes (*FGFR1*, *FGFR2*, *FGFR3*, and *FGFR4*) and *FGFRL1*, a truncated FGFR without intracellular domain,^2^ in mammals. FGFRs are single-pass transmembrane proteins containing an extracellular domain, a transmembrane domain (TMD), and an intracellular tyrosine kinase domain. Among them, the extracellular domain is composed of three immunoglobulin (Ig)-like domains (D1–D3), an acidic region, a heparin-binding motif for FGFs, heparan cofactors, and partner proteins. The TMD anchors the receptors in cell membrane and facilitates its dimerization. In the cytosol, the juxtamembrane region of FGFRs is involved in receptor dimerization, while the split kinase domains are required for the transmitting of FGF-related signaling.^3^

**Fig. 2:**
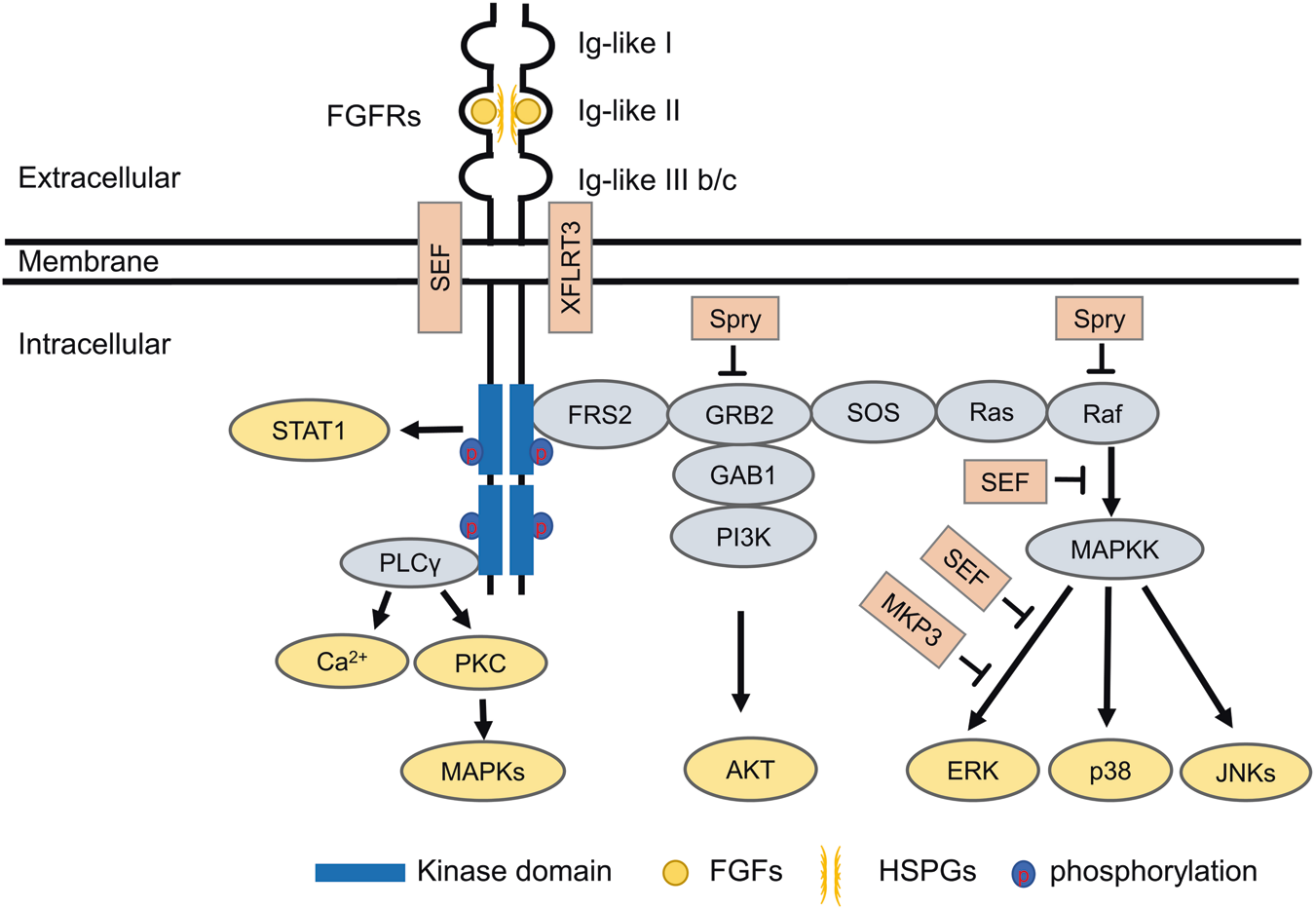
The classical FGF/FGFR pathways. Binding of appropriate growth factors to receptors triggers the conformational changes of FGFRs, resulting in dimerization and activation of FGFRs. Activated FGFRs phosphorylate FRS2a and FRS2a binds to SH2 domain-containing adaptor Grb2. Grb2 will subsequently bind to SOS, GAB1, and Cbl through its SH3 domain to activate Ras/Raf/MAPKs, including ERK MAPK, p38 MAPK, and JNK MAPK. The activated FGFRs also activate phosphatidylinositol (PI)-3 kinase and STAT. FGFRs recruit and phosphorylate PLCγ. Among the members of the FGF synexpression group, SEF and XFLRT3 are transmembrane proteins and can interact directly with FGFRs. SEF functions as a negative regulator by affecting the phosphorylation of the MAPK ERK cascade. XFLRT3 forms a complex with FGF receptors and enhances FGF/FGFR signaling. Spry acts at the level of Grb2 and/or the level of Raf to attenuate FGF/FGFR signaling. MKP3 negatively regulates FGF/FGFR signaling by dephosphorylating the activated ERK. FRS2α FGFR substrate 2α, GAB1 GRB2 associated binding protein 1, GRB2 growth factor receptor-bound 2, PKC protein kinase C, SOS son of sevenless

The binding of FGFs to the inactive monomeric FGFRs will trigger the conformational changes of FGFRs, resulting in dimerization and activation of the cytosolic tyrosine kinases by phosphorylating the tyrosine residues within the cytosolic tail of FGFRs.^4^ Then, the phosphorylated tyrosine residues serve as the docking sites for downstream signaling molecules, such as FGFR substrate 2α, which is localized on the plasma membrane.^5^ FGFRs also recruit and phosphorylate SH2 domain-containing substrate phospholipase Cγ (PLCγ) by formatting an allosteric 2:1 FGFR–PLCγ complex, indicating that FGFR dimerization plays an obligatory role in substrate phosphorylation.^6^ Depending on the cellular content in distinct cells and tissues, the classical FGF/FGFR downstream signaling pathways include Ras/Raf-MEK-MAPKs (mitogen-activated protein kinases), phosphatidylinositol-3 kinase/protein kinase B (PI3K/AKT), PLCγ, and signal transducer and activator of transcription (STAT).^1,7^ Additionally, several proteins belonging to FGF synexpression group have been identified, such as Sprouty (Spry),^8,9^ XFLRT3,^10^ SEF,^11,12^ MKP3,^13,14^ and so forth. These proteins are themselves regulated by FGF signaling and are tightly co-expressed with FGFs. Most of them inhibit FGF/FGFR signaling by establishing negative feedback loops^15^ (Fig. 2).

The diversified functions of FGF/FGFR signaling indicate the complex regulation of the signaling cascades. FGF/FGFR signaling can be modified at several levels, including ligand–receptor binding specificity,^16^ expressions^1^ and alternative splicing,^17^ and the crosstalk between FGFs/FGFRs and other signaling cascades,^18^ such as BMP (bone morphogenetic protein)^19^ and Wnt signalings.^20,21^ FGF/FGFR binding specificity/promiscuity combined with ligand-dependent differences in receptor orientation is the main mechanisms for the precise regulation of FGF-induced signaling.^16^ FGF/FGFR signaling is tightly regulated by the spatial and temporal expressions of FGFs, FGFRs, and heparan sulfate cofactors.^15,22^ Diversified tissue distribution and different expression levels of signaling components, which influences the function of FGF/FGFR signaling, eventually affect the tissue development, maintenance, and disease pathogenesis.^1^ Alternative splicing and translational initiation generate multiple isoforms of FGFs/FGFRs and regulate their expression levels.^23^ For example, the tissue-specific alternative splicing in D3 of FGFR1, FGFR2, and FGFR3 can generate b and c isoforms, and thus determines the binding specificity/promiscuity for individual FGFs at diverse cells and tissues.^24^ Furthermore, it is well documented that epigenetic mechanisms,^25^ the posttranslational modifications, such as phosphorylation,^26^ glycosylation,^27^ ubiquitination,^28^ and cellular trafficking of FGFs/FGFRs^29,30^ are also involved in the regulation of the expressions of FGF/FGFR signaling components and the signal specificity, intensity, and timing.

During the past decades, repaid progresses have been made about the modulation of FGF/FGFR signaling cascades; these studies not only deepen our understanding of the unique properties of FGF/FGFR signaling, but also raise the opportunity for developing new therapies targeting causative FGF/FGFR signaling.

### Coreceptors of FGFs/FGFRs

Usually, specific ligands require assembly of the ternary complexes composed of ligand, receptor, and coreceptor at the cell surface to initiate signal transduction. The coreceptors of FGF/FGFR cascade include heparan sulfate proteoglycans (HSPGs) (for paracrine FGFs) and Klotho (for endocrine FGFs).

#### HSPGs

HSPGs are glycoproteins, containing one or more covalently attached heparan sulfate (HS) chains. According to their location, the HSPGs are grouped into three groups: membrane HSPGs, such as syndecans and glycosylphosphatidylinositol-anchored proteoglycans (glypicans), the secreted extracellular matrix HSPGs (agrin, perlecan, type XVIII collagen), and the secretory vesicle proteoglycan, serglycin.^31^ HSPGs is a mandatory cofactor in paracrine FGF signaling. Paracrine FGFs have moderate to high affinity for HSPGs, which shortens FGF diffusion distance away from their secretion cells. The interaction also provides a depot of regulatory factors that can be released by selective degradation of the HS chains facilitating the formation of FGF gradients essential for cell specification during development and regeneration.^22^

Structural studies have revealed that the HSPG binding site of FGFs contains the β1–β2 loop and the extended β10–β12 region, and each FGF ligand has discrete affinity for HSPGs.^32^ HSPG-mediated FGF-specific morphogenetic gradients contribute to the distinct function of FGFs. Importantly, endocrine FGFs such as FGF19 and FGF23 lack the paracrine-conserved glycine box and the truncated β10–β12 region in the potential HS binding region, reducing the binding affinity between HSPGs and the endocrine FGFs (FGF19 subfamily), which allows these FGF ligands to permeate through the HSPG-rich extracellular matrix (ECM) and subsequently enter the blood circulation.^33^

Detailed crystal studies reveal that HSPGs promote the formation of a 2:2:2 dimer between FGF, FGFR, and HSPGs.^34^ By engaging ligand and receptors in the dimer, HSPGs promote the kinetics and thermodynamics of FGF-FGFR binding and dimerization, which is required for the transmission of a sustained and robust intracellular signals.^34^

#### Klotho

Klotho are coreceptors for endocrine FGF signaling. As single-pass transmembrane proteins, Klotho consists of tandem KL domains, and are homologous to β-glucosidases. Modeling studies showed that the endocrine FGFs (FGF19, FGF21, and FGF23) exhibit a negligible HSPGs binding affinity and poor affinity for their cognate FGFRs, resulting in ineffective endocrine FGF/FGFR binding and dimerization.^33^ It is well established that α/β Klotho coreceptors are required for these ligands to initiate respective signaling activity.^33^ The Klotho coreceptors associate constitutively with the c-splice isoforms of FGFR1-3 and FGFR4 to promote their binding of FGFs and dimerization, reinforcing FGF/FGFR signaling specificity. For example, FGF23 can bind and activate FGFR1c-α-Klotho, FGFR3c-α-Klotho, and FGFR4-α-Klotho. A recent atomic structure study showed that α-Klotho simultaneously binds FGFR1c and FGF23, and dimerization of the stabilized ternary complexes and receptor activation depend on the binding of HS.^35^ FGF19 activates FGFR1c-β-Klotho (KLB) and FGFR4-KLB, whereas FGF21 mainly activates the FGFR1c-KLB complex.^36^

Endocrine FGF/FGFR signaling rely on the interaction between FGFRs and Klothos. Biochemical studies revealed that α-Klotho combines with FGFR1c to create a de novo site for the FGF23 carboxy tail, whereas KLB uses two distinct sites to independently bind FGFR and the carboxy tail of FGF19 or FGF21.^37,38^ The proteolytically cleaved FGF23 carboxy tail can competitively inhibit the binding of native FGF23 to the FGFR1c-α-Klotho complex and thus downregulate FGF23 signaling.^39^ In patients with autosomal-dominant hypophosphatemic rickets (ADHR), the mutations in the RXXR motif located in the carboxy tail abrogate the proteolytic cleavage of FGF23 and thus elevate the serum levels of full-length bioactive FGF23, which accelerates the excretion of phosphate from the kidney.^40,41^ Mutations in D3 hydrophobic groove of FGFRc isoforms and FGFR4 residues abolishes Klotho binding, indicating the overlapping between FGFs and Klotho binding sites on FGFRs.^38^ The association of FGFRs with the Klotho coreceptor decreases the ability of these receptors to respond to paracrine FGFs, such as FGF8, supporting the notion that endocrine and paracrine FGF signaling affect each other.^38^

### Modulators of FGF/FGFR signaling

#### Cell adhesion molecules (CAMs)

CAMs are typically single-pass transmembrane receptors and include four major groups: cadherins, integrins, the Ig superfamily of CAMs (IgCAMs), and the superfamily of C-type of lectin-like domains proteins.^42^ A growing body of data reveals that various CAMs can act as FGFR binding partners, participating in the modulating of FGF/FGFR signaling and are strongly implicated in cell fate determination of different cell lineages.^43^

Cadherins play an essential role in the formation and adaptive reinforcement of adherens junctions, and modulation of the dynamics of actin cytoskeleton.^44^ Different members of the cadherin family are expressed in a cell type-specific manner, and most of the cell types express multiple cadherins, including VE-, N-, and T-cadherin. N-cadherin is associated with FGFRs through their acidic box-mediated activation of FGFRs and their downstream signaling in numerous cells.^45^ In breast cancer cells, formation of N-cadherin complexes with FGFR1 can decrease the internalization and lysosomal degradation of FGFR1, and thus sustain the receptor signaling via MAPKs, whereas silencing of N-cadherin results in the accelerated FGFR1 degradation. Thus, N-cadherin stabilizes FGFR1 and simultaneously enhances FGF2-induced proliferation and differentiation of epiblast stem cells.^46^ In addition, cadherin-11–FGFR1 interaction occurs through their extracellular domains. Cadherin-11 initiates intracellular signaling pathways via FGFR1 and recruits FGFR1 into the cell–cell contact area. The cadherin-11-induced FGFR1 signaling stimulates neurite outgrowth.^47^

The FGFR/neural CAM (NCAM) complexes have been observed in multiple cell types.^48^ The FN3 domains of NCAMs mediate its interaction with the Ig2–Ig3 region of FGFRs.^49^ NCAMs bind to FGFR1–FGFR3 to activate the receptor and initiation of signaling cascades and inhibit FGFR K27- and K29-linked polyubiquitination and lysosomal degradation.^50^ Interestingly, NCAMs can affect the cellular trafficking of FGFRs.^51^ In contrast to FGF-induced activation and lysosomal degradation of endocytic FGFR1, NCAM can promote the stabilization of FGFR1, which is recycled from endosomes to the cell surface through a Rab11 and Src-dependent manner.^51^

Integrins act as the receptors for extracellular matrix molecules, playing a key role in regulating intercellular contact and intracellular signaling. Eighteen α-subunits and eight β-subunits assemble into 24 functional integrins that vary in terms of ligand specificity and cellular function.^52^ Each α–β combination can bind to unique matrix components. Increasing evidences showed that integrins modify FGF/FGFR signaling.^53^ For example, the fibronectin-binding α5β1-integrin dimer upregulates FGF2 expression, while secreted FGF2 directly binds to αvβ3 integrin.^54,55^ FGF1, FGFR1 and integrin αvβ3 can be assembled into a ternary complex, in which FGF1 acts as a bridging molecule, to maintain sustained activation of FGFR1-dependent kinases ERK1/2.^56^

NCAM is a member of IgCAMs containing Ig-like and fibronectin type III (FNIII) domains. NCAM plays a critical role in neurite outgrowth as binding partners affecting the signaling process. A peptide derived from the NCAM FNIII region binds to FGFR1 directly to stimulate FGFR1 phosphorylation in primary rat neurons.^51^ In PC12 cells, NCAM requires FGFRs to promote neurite growth.^57^ Specifically, the NCAM-FGFR interaction activates PLCγ and diacylglycerol lipase to generate arachidonic acid, elevating intracellular calcium levels and activating Ca^2+^-dependent protein kinase C (PKCs).^58^ NCAM has been found to form a complex with FGFR4. This complex can lead to β1-integrin-mediated cell–matrix adhesion, and also decrease the mobility of pancreatic tumor cells by stimulating FGFR4 kinase activity.^59^

#### G protein-coupled receptors

G protein-coupled receptors (GPCRs) constitute the largest groups of receptors that mainly transmit various signals across cell membranes through binding and activating heterotrimeric G proteins. Structurally, GPCRs are composed of an N-terminal extracellular domain, seven-transmembrane helices, and a C-terminal region.^60^ A growing number of studies have revealed that various members of GPCRs and receptor tyrosine kinase (RTKs) can form heterocomplexes together and trigger different intracellular signaling and cellular response.^61,62^ The GPCRs can transactivate multiple RTKs,^63^ including epidermal growth factor receptor,^64^ platelet-derived growth factor receptors (PDGFRs),^65^ and insulin-like growth factor receptors,^66^ and so on.

In the central nervous system, both GPCR and FGFR signaling are involved in the control of proliferation, migration, survival, and differentiation of neurons. More and more studies have showed that GPCRs form heterocomplexes with FGFRs and regulate the cell fate of neurons.^67^ Multiple methods have confirmed the interaction between FGFR1 and adenosine receptor A2AR. The function study revealed that this interaction is required for the enhanced activation of ERK1/2, which is important for the regulation of the synaptic plasticity.^68^ Another study showed that cannabinoid receptor 1 (CB1R)-FGFR1 complexes occur in the lipid rafts of the plasma membrane, leading to activation of ERK1/2, and play important roles in neuronal differentiation.^69^ CB1R activates Fyn and Src via PKC signaling, inducing the transactivation of FGFR1 by phosphorylating its kinase domain.^69^ The interactions between FGFR1 and muscarinic acetylocholine receptor (mAChR) subtype M1R and 5-hydroxytriptamine receptor 1A (5-HT1A) have been visualized.^70^ Stimulation of hippocampal neurons with M1R agonist oxotremorine-M activated FGFR1, and the crosstalk between mAChR and FGFR1 enhanced the neurite growth.^71^ Treatment of FGF2 and 5-HT1A agonist 7-(dipropylamino)-5,6,7,8-tetrahydronaphthalen-1-ol (8-OH-DPAT) can increase the FGFR1–5-HT1A complexes; activation of 5-HT1A by 8-OH-DPAT causes subsequent FGFR1 phosphorylation mediated by Src.^70^ Interestingly, the FGFR1-5-HT1A heterocomplexes display anti-depressive effects and thus may be the novel targets for the treatment of mood disorders.^72^

#### Other RTKs

FGF/FGFR signaling can also be modified by their interplay with other members of RTK family. The crosstalk among RTKs can occur at different levels, such as the ligand, receptor, and downstream cascades. Among them, different RTKs can form receptor heterocomplexes and subsequently cause tyrosine phosphorylation of one receptor by tyrosine kinase of the other one. Binding with other RTKs gives another way to modify FGF/FGFR activities more elegantly.

Eph receptors constitute the largest family of RTKs, including EphA (EphA1-EphA10) and EphB (EphB1-EphB6) receptors, and are activated by ephrin ligands.^73^ The Eph receptors contain structural features characteristic for RTKs. The Eph receptor-ephrin complexes regulate cell adhesion, organization of cytoskeleton, angiogenesis, neural development, and plasticity.^74^ EphA4 receptor interacts with FGFRs through the tyrosine kinase domain of Eph4 and the JM domain of FGFR1-4.^75^ More detailed analysis revealed that phosphorylation of the tyrosine residues within JM domain of Eph4 is required for the formation of EphA4–FGFR complexes. Kinase domains of EphA4 and FGFRs can trans-phosphorylate each other.^75^ Importantly, the ternary complex, involving FGFR1, EphA4, and FRS2α, was detected. FRS2α may act as a tethering molecule that integrates signals from both receptors and regulates the self-renewal, proliferation, and differentiation of neural stem/progenitor cells.^76^ Studies also showed that FGFR phosphorylate ephexin1, a targeting molecule of EphA receptors.^77^ Scaffolding protein Dlg-1, which directly interacts with EphA receptors, can also modulate FGFR signaling.^78^

PDGFRα and PDGFRβ are activated by multiple PDGFs: PDGF-AA, PDGF-BB, PDGF-AB, PDGF-CC, and PDGF-DD.^79^ PDGFR-mediated signaling can regulate cell motility, proliferation, angiogenesis, and are involved in a range of diseases.^80^ In vitro and in vivo experiments revealed that both PDGFRα and PDGFRβ interact with high affinity with FGFR1.^81^ The formation of PDGFRα-FGFR1 complexes is facilitated by the presence of ligands for both receptors. In receptor heterocomplex, PDGFRβ can directly phosphorylate FGFR1 on tyrosine residues.^81^ Interestingly, FRS2α functions as a bridging molecule between PDGFRβ and FGFR1, further supporting the speculation that FRS2α may act as a tethering molecule integrating signals from different RTKs.^81^

### Nuclear FGFs and FGFRs

In addition to the FGF/FGFR complexes at plasma membrane, it has been recognized that canonical FGF ligands and FGFRs can enter the nucleus of multiple types of cells and tissues.^82^ Nuclear localization of FGFs/FGFRs lends an additional layer of regulatory complexity.^83,84^ Nuclear FGFs/FGFRs can exert their effects on proliferation, lineage commitment, and gene expressions. Dysregulation of nuclear FGFs/FGFRs has been found in congenital skeletal disorders and neoplastic transformation.^85^

Nuclear localization of FGFs and FGFRs has been demonstrated in multiple tissues in different pathophysiological conditions. During gonadal development, FGFR2 is firstly localized to the plasma membrane of proliferating sertoli progenitor cells, but in the early stage of specification and differentiation, FGFR2 is colocalized with SRY and SOX9 in the nucleus of sertoli cell.^86^ In the development of salivary gland, nuclear FGFR2 is specifically located in proliferating epithelial cells at the branch tips in response to FGF10.^87^ In human pancreatic cancer cells, FGFR1 and FGF2 are localized to the nucleus where they promote proliferation and invasion.^88^ In breast mucinous carcinoma, nuclear FGFR2 is commonly found colocalized with STAT5 and Runx2.^89^ The nuclear FGFR3 levels in breast, bladder, and pancreatic cancer cells are higher than those in corresponding non-tumor tissues.^90^

Several FGF ligands contain a nuclear localization signal to facilitate their nuclear import, and different mechanisms are involved in the receptor nuclear localization.^91,92^ In some cases, nuclear localization of full-length FGFRs occurs through a ligand-dependent mechanism. For example, FGF2, FGF1, and FGF10 localize to the nucleus with FGFR1.^93,94^ Structurally, all FGFRs contain a single-pass TMD, the major determinant of intracellular localization. Mutations in the TMD in FGFR1 and FGFR2 remarkably affect their subcellular distribution. FGFR2 mutations (FGFR2^M391R^ and FGFR2^Y381D^) located in the TMD can reduce plasma membrane levels of FGFR2, and amplify its nuclear and nucleolar presence in growth plate chondrocytes derived from patients with skeletal disorder bent bone dysplasia syndrome (BBDS).^95,96^ Interestingly, posttranslational modifications, such as glycosylation, also contribute to the nuclear localization of FGFRs. In the skeletal disorder Crouzon syndrome, the FGFR2 mutation (FGFR2^C278F^) leads to incomplete FGFR2 glycosylation, blocks its membrane localization, and induces the perinuclear accumulation of receptor.^97^ It was found that FGFR1 and FGFR2 exert their nuclear import through a β-importin-dependent active nuclear pore-mediated mechanisms,^93^ and proteolytically cleaved FGFR1 and FGFR3 mediated by granzyme B and γ-secretase localize in the nucleus of invading cancer cells and multiple cell lines,^94^ but the detailed molecular events are still unclear.

Once in the nucleus, FGFs and FGFRs can promote gene expressions through multiple approaches, such as epigenetic mechanisms. In embryonic stem cells and neuronal cells, FGFR1 binds the proximal promoters and activates the transcription of pluripotency-related genes, Wnt/β-catenin signaling components, and P53.^98^ In preosteoblasts, FGFR2 and FGF2 localize to the nucleolus to recruit histone remodeling factors, such as the CBP homolog p300, to ribosomal DNA (rDNA) and activate RNA polymerase I-mediated transcription, increasing ribosome biogenesis and subsequently protein synthesis.^95,96^ Nuclear FGF/FGFR-mediated regulation of transcription suggests an alternative way through which FGFs/FGFRs can directly induce specific and rapid changes of gene expressions. In osteoprogenitor cells, nuclear FGFR2-mediated regulation of rDNA transcription promotes self-renewal over terminal osteoblast differentiation.^95,96^ In invading breast cancer cells, FGFR1 undergoes nuclear translocation and activates the transcription of genes critical for cell migration.^94^ The activating mutant FGFR2 Y376C in endometrial cancer has increased perinuclear localization and appears to be involved in disrupting cell polarity in metastatic cells.^99^ In pancreatic cancer, nuclear FGFR3 correlates with metastatic disease and poor overall prognosis.^90^

Compared with the well-established mechanisms in transmembrane signaling, the mechanisms for FGF/FGFR cascades in the nucleus are less studied. Nuclear localization of RTKs is not unique to the FGFs/FGFRs.^100,101^ It is very important to clarify the precise mechanisms for nuclear FGFR translocation, activation of downstream pathways, and target genes, as well as its functions in different pathophysiological conditions in the future study.

## FGF signaling in skeleton development and repair/regeneration

### Expressions of FGFs and FGFRs during skeleton development

Both FGFs and FGFRs have characteristic spatiotemporal expression patterns throughout all stages of skeletal development (Table 1).^102^

**Table 1:**
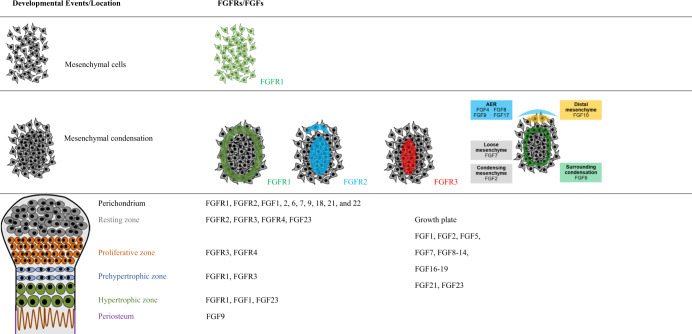
FGF and FGFR expression in long bone development

During limb bud development, the active epithelial–mesenchymal interactions between ectoderm-expressed FGF (FGF8) and FGFR2b, and the mesenchyme-expressed FGF (FGF10) and FGFR1c, are indispensable for the outgrowth and patterning of limbs.^103^ *FGFs* *4*, *8*, *9*, and *17* are specifically expressed in the mouse apical ectodermal ridge (AER), a major signaling center at the distal edge to ensure proper development of limb buds. FGF9 is located in regions corresponding to mesenchymal condensations in AER,^104^ and is only expressed in the mesenchyme surrounding the cartilaginous condensations at the later stage. FGF9 is then expressed in the perichondrium/periosteum and primary spongiosa.^105^ In rat, Lazarus et al.^106^ found that *FGFs 1, 2, 6, 7, 9, 18, 21*, and *22* are expressed in the perichondrium, while FGFs 2, 7, 18, and 22 are expressed in the growth plate. FGFs 1, 2, 17, and 19 are the predominant FGF ligands expressed in human fetal growth plate cartilage.^107^ *FGF18* is expressed in the periosteum, the articular surface, synovial tissue, and in cells within the perichondrial groove of Ranvier.^108^ During intramembranous bone formation, *FGF8* is expressed in developing calvarial osteoblasts, *FGF9* is expressed in calvarial mesenchyme, and *FGF18* is expressed in mesenchymal cells and differentiating osteoblasts, whereas *FGF23* is mainly produced by differentiated osteoblasts and osteocytes.^109^

*FGFR1* and *FGFR2* are existed in mesenchymal cells prior to morphological indication of mesenchymal condensation. *FGFR1* is evenly expressed in limb bud mesenchyme, while the expression of *FGFR2* is increased in chondrogenic condensation area, as the first marker of chondrogenic condensation. Both *FGFR1* and *FGFR2* are expressed in the periphery of the condensation, where is the location of the origin cells of perichondrium and periosteum.^109^ In the established growth plates, FGFR3 is expressed mainly in the resting, proliferating, and prehypertrophic zone.^110–112^ As chondrocytes begin to hypertrophy, *FGFR3* expression is shut down, while the expression of *FGFR1* is elevated. It has also been found that FGFR2 is expressed in the resting zone, while FGFR4 is expressed in the resting and proliferative zones.^106^ *FGFR3* is expressed more intensely in latent chondroprogenitor cells located in the groove of Ranvier and ring of LaCroix.^113^ The expressions of FGFR1 and FGFR2 in osteoblasts have been well characterized.^112^ FGFR3 is also found expressed in osteoblasts.^114,115^ In cranial sutures, FGFRs are expressed in a spatial-dependent manner. FGFR2 is predominantly expressed in osteoprogenitor cells, while FGFR1 is located in more differentiated osteoblasts.^116^ FGFR3 has lower expression in the periosteum and sutural osteogenic fronts at the late stage of suture development.^117^

### FGF/FGFR-related genetic diseases with abnormal skeleton development in humans

The characteristic expression patterns of FGFs/FGFRs imply the critical roles of FGFs/FGFRs in skeletal development, and both gain-of-function (GOF) and loss-of-function (LOF) mutations in individual FGFRs or FGFs have been found to cause a variety of genetic skeletal diseases in humans.

Mutations and single-nucleotide polymorphisms (SNPs) of *FGFs* have been linked to multiple skeletal disorders. Constitutionally increased dosage of *FGF3* and *FGF4* genes is a risk factor of craniosynostosis.^118^ Heterozygous mutation in *FGF3* gene causes deafness, congenital inner ear agenesis, microtia, and microdontia.^119^ Heterozygous mutation of *FGF8* can lead to autosomal-dominant hypogonadotropic hypogonadism-6 with or without anosmia characterized by short stature, hyperlaxity of the digits, camptodactyly, and mild scoliosis.^120^ FGF8 mutation also accounts for a small percentage of Kallmann syndrome (KS).^121^ FGF9 heterozygous missense mutations S99N and R62G have been identified to be responsible for multiple synostoses syndrome 3, and some individuals showed sagittal suture synostosis and humeroradial synostoses in humans.^122,123^ LOF mutations in FGF10 cause an autosomal-dominant multiple congenital disorder characterized by lacrimal duct aplasia, malformed ears and deafness, and disturbed distal limb segments, named lacrimo-auriculo-dento-digital syndrome.^124^ FGF10 is identified as a genetic risk factor for nonsyndromic cleft lip with or without cleft palate.^125^ Truncated mutations of FGF16 are associated with X-linked recessive hand malformations with metacarpal 4/5 fusion.^126^ Congenital hypogonadotropic hypogonadism individuals caused by missense mutations of FGF17 displayed low bone mass.^127^ Missense mutations such as R176Q, R179W, and R179Q in FGF23 cause ADHR, frequently present with rickets, bone pain, and tooth abscesses.^128^ LOF mutations in *FGF23* cause a rare autosomal recessive metabolic disorder, hyperphosphatemic familial tumoral calcinosis, characterized by the progressive ectopic calcifications and elevated serum phosphate levels.^129^

A GOF missense mutation in FGFR1 (P252A) leads to Pfeiffer syndrome (PS), a craniosynostosis syndrome with characteristic abnormalities, including broad thumbs and toes, brachydactyly or variable syndactyly, and elbow ankylosis.^130,131^ Several FGFR1 mutations, such as N330I and C379R, result in osteoglophonic dysplasia (OGD), characterized by craniofacial abnormalities, including craniosynostosis and depressed nasal bridge, rhizomelic dwarfism, and non-ossifying bone lesions.^132^ LOF mutations such as C277Y, R622X, and A167S in FGFR1 are responsible for autosomal-dominant KS, characterized by hypogonadotropic hypogonadism and anosmia. Some KS cases present skeletal abnormalities, such as scoliosis, limb anomalies, and loss of nasal cartilage.^133^ GOF mutations of FGFR2, mainly in the third Ig-like domain and adjacent linker regions (exons IIIa and IIIc), lead to multiple types of autosomal-dominant craniosynostoses, such as Apert syndrome (AS), Crouzon syndrome, and PS, as well as Beare-Stevenson cutis gyrata syndrome.^134–138^ Several de novo missense mutations of FGFR2 have been identified responsible for a perinatal lethal skeletal dysplasia entitled as BBDS-FGFR2 type characterized by deformities in multiple bone, including mineralization disorder of the calvarium, craniosynostosis, and dysmorphic facial features, as well as bent long bones and osteopenia.^139^ GOF mutations in FGFR3 affect predominantly bones developed through endochondral ossification causing hypochondroplasia, achondroplasia (ACH), and thanatophoric dysplasia (TD, type I/II).^140,141^ GOF mutations in FGFR3 have also been found to cause craniosynostoses. The A334T mutation of FGFR3 cuases mild craniosynostosis,^142^ while A391E mutation in FGFR3 TMD is responsible for Crouzon syndrome with acanthosis nigricans.^143^ FGFR3 P250R and P252R mutations cause Muenke syndrome, an autosomal-dominant disorder characterized by uni- or bi-coronal synostosis, macrocephaly, midfacial hypoplasia, and developmental delay.^144^ Some TD patients exhibit joint fusion and craniosynostoses.^145^ FGFR3 with R621H substitution in the tyrosine kinase domain and a homozygous missense mutation-T546K, leading to partial loss of FGFR3 function, cause camptodactyly, tall stature, and hearing loss syndrome.^146,147^ To date, no mutation of FGFR4 has been found responsible for genetic skeletal disorders in humans.

### FGF/FGFR signaling in skeleton development and homeostasis

Accumulating studies dissecting the roles of FGFs/FGFRs in the development and homeostasis of skeleton have been carried out by using animal models and cell/tissue culture systems.

#### FGFs in skeleton development and homeostasis

FGF1 has been shown to play an important role in regulating the fate of bone marrow stromal cells (BMSCs) by inhibiting osteogenesis and promoting adipogenesis.^148^ FGF2 is expressed in osteoblasts and the stromal cells in the bone. Stored in the extracellular matrix, FGF2 promotes both osteoblastic and chondrogenic differentiation of cranial neural crest cells.^149^ Mice with non-targeted overexpression of FGF2 show shortened long bones caused by premature closure of the epiphyseal plate.^150^ Sobue et al.^151^ found that overexpression of FGF2 in mice leads to osteopenia and defective mineralization, proposing that FGF2 functions as a negative regulator of bone formation. The roles of the nuclear high molecular weight (HMW FGF2) and secreted low molecular weight (LMW FGF2) isoforms have been well clarified. The HMW FGF2 has an inhibitory effect on bone mineralization, while the LMW FGF2 promotes bone formation through the regulation of Wnt, BMP2, FGF23, and phosphate homeostasis.^152,153^ In the articular cartilage, FGF2 binds to perlecan in the pericellular matrix and acts as a mechanotransducer.^154^ Full-length FGF2 or LMW FGF2 ablation in mice leads to early onset of osteoarthritis (OA), whereas loss of HMW FGF2 isoform has a protective effect on the articular cartilage.^155^ FGF2 can upregulate the transcription of matrix metallopeptidases 1 and 13 (MMP1 and MMP13), stimulate ADAMTS 5 expression,^156–158^ and accelerate matrix degradation via a neuro-endocrine pathway in human adult articular chondrocytes.^159^ FGF3 together with BMP signaling regulates the specification of neural crest and the extension of anterior-posterior axis.^160^ FGF signaling (FGF3 and FGF8a) together with SHH hierarchically regulates the early specification of skull in zebrafish.^161^ FGF4 has been shown to be involved in the development and axial elongation of embryonic murine^162,163^ and Kratochwil et al.^164^ concluded that FGF4 is a direct target of Wnt signaling during tooth development in mice. FGF6 signaling transduction is mainly mediated by FGFR1 (osteoblasts and osteoclasts) and FGFR4 (osteogenic precursor cells and osteoblasts), which can activate RANKL (receptor activator of nuclear factor-κB (NF-κB)) to stimulate osteoclasts.^165^ FGF8 participates the regulation of osteogenic and chondrogenic fate in mesenchymal cells in the skull and hard palate.^166,167^ Hung et al.^105^ revealed that FGF9 can promote the hypertrophy of chondrocytes and regulate vascularization in growth plates. Transgenic overexpression of FGF9 in mouse chondrocytes led to decreased proliferation and terminal differentiation of chondrocytes, which mimics the phenotype of ACH.^104^ FGF9 is required for the normal expression of Gdf5 in the prospective joints through the regulation of Gdf5 promoter activity.^168^ *FGF10* is present in the osteoprogenitors in condensation region of the frontal bone, and genetic knockdown (KD) of *FGF10* can partially rescue the skeletal phenotype such as craniosynostosis and sternal abnormality in AS mouse model.^169^ FGF11 is involved in the simulation of osteoclast-mediated bone resorption induced by hypoxia.^170^ FGF17 can inhibit the proliferation of FGFR3-expressing rat chondrosarcoma chondrocytes.^107^ FGF18-deficient mice show delayed suture closure with decreased proliferation and delayed osteogenic differentiation of calvaria osteogenic mesenchymal cells, and increased proliferation and differentiation of chondrocytes, indicating that FGF18 positively regulates proliferation and differentiation during osteogenesis, while acts negatively in chondrogenesis.^171,172^ It has been reported that the deformities of the calvaria, ribs, hindlimb, forelimb, and axis in mice with mesenchyme-specific *FGF18* inactivation are dependent on the expression of *FGF18* originating from the mesenchymal compartment.^108^ Serum FGF21 concentration is positively correlated with lumbar BMD.^173^ FGF21 can lead to growth attenuation by antagonizing the stimulatory effects of growth hormone and even directly suppress the proliferation and differentiation of chondrocytes in the growth plate.^174^ FGF21 can enhance the osteogenic effect of BMP2.^175^ In addition, FGF21 is essential for lactation-induced skeletal changes.^176^ Transgenic mice with overexpression of *FGF23* exhibit short stature, lower extremity deformities, and osteomalacia with low serum phosphate concentration.^177^ Conversely, *FGF23*-deficient mice exhibit hyperphosphatemia, ectopic mineralization, and poorly formed skeleton with an extremely low parathyroid hormone (PTH) level and elevated 1,25-dihydroxyvitamin D3 (1,25(OH)_2_D_3_) level in the serum.^178^ FGF23 can suppress chondrocyte proliferation through suppression on *IHH* expression.^179,180^ FGF23 secreted from osteocytes may regulate mineralization through FGFR3 in a 1,25(OH)_2_ D_3_ and Klotho-independent manner via an autocrine/paracrine feedback loop.^181^

#### FGFRs in skeletal development and homeostasis

The roles of FGFRs in skeletal development and especially in genetic skeletal diseases have been further dissected by employing genetically modified animal models.

Zhou et al.^182^ found that mice carrying a P252R mutation in FGFR1 can mimic human PS with premature fusions of multiple sutures, accelerated osteoblast proliferation, and increased expressions of osteogenic genes, and further uncovered that *CBFA1* may be a downstream target of FGF/FGFR1 signals in vitro. Trokovic et al.^183^ concluded that FGFR1 is expressed in pharyngeal region and create a permissive environment for neural crest cell migration in mice homozygous for a hypomorphic allele of *FGFR1* with craniofacial defects. The hush puppy FGFR1 W691R mutation is unresponsive to FGF1 in calcium mobilization and downstream signaling through MAPK or PLCγ and can lead to ear defects and skull abnormalities in mice.^184^ By deletion of *FGFR1* in osteochondro-progenitor cells and differentiated osteoblasts in mice, it is proposed that FGFR1 promotes the differentiation of mesenchymal progenitors into osteoblasts, but inhibits the maturation and mineralization of osteoblasts.^112^ Mice lacking *FGFR1* in chondrocytes showed shortened stature and tibial length with expanded hypertrophic zone in growth plate, indicating the important role of FGFR1 during chondrocyte maturation.^185^ FGFR1 signaling in mature osteoblasts/osteocytes is required for the survival of osteocytes and bone mass maintaining in mice.^186^ In addition, our group revealed that FGFR1 can positively regulate the differentiation and resorption activity of osteoclasts.^187^

GOF mutation in FGFR2 (S252W) resulted in increased apoptosis of osteogenic cells,^188^ disturbed osteoblastic proliferation and differentiation, and the presence of ectopic cartilage at the midline sagittal suture.^189^ We observed that FGFR2-P253R mutation can directly affect both intramembranous and endochondral ossification in mice.^190,191^ Cells isolated from limbs of mice with FGFR2 S252W mutation can differentiate into chondrocytes in the osteogenic medium, suggesting that FGFR2 may affect the fate of mesenchymal cells.^189^ Further studies on BBDS resulting from FGFR2 mutations revealed that nuclear FGFR2 regulates the developing limb, musculoskeletal integration, and cell fate determination.^96,192^ Targeted disruption of FGFR2IIIc in mice leads to narrowed proliferative and hypertrophic zones in growth plate, and disturbed ossification with downregulation of *IHH*, *PTHRP*, and *RUNX2*.^17^ Yu et al.^193^ found that conditional deletion of FGFR2 in mesenchyme can lead to skeletal dwarfism and decreased bone mineral density with dramatically disturbed proliferation of osteoprogenitors and anabolic function of mature osteoblasts in mice. In zebrafish, *FGFR2* is essential for the mesenchyme condensation, later chondrogenic differentiation, and survival of chondrocytes in late cranial cartilage development.^194^

Mice with FGFR3 mutation mimicking human ACH and TD II exhibit dome-shaped skulls and chondrodysplasia,^195,196^ while *FGFR3* deficiency in mice causes increased bone length,^197,198^ indicating that FGFR3 is a negative regulator of endochondral bone formation. The expression levels of P16, P19, and P21 are upregulated in growth plates of ACH mice and FGF2 treatment can stimulate the expressions of P21 and P27 in RCS cells,^195,199,200^ suggesting that the upregulation of cell-cycle inhibitors may be involved in activated FGFR3-induced growth arrest of chondrocytes. FGFR3 downregulates PTH/PTHrP (PTH-related peptide) signaling partially through the Janus kinase/STAT pathway.^201–203^ Reduced telomerase activity may participate in the inhibitory effect of FGFR3 on the proliferation of chondrocytes.^204^ There are contradictories about the role of FGFR3 in the differentiation of chondrocytes. *FGFR3* deficiency in mice causes enhanced chondrocyte hypertrophy;^197,198^ activated FGFR3 inhibits the hypertrophic differentiation of chondrocytes in cultured metatarsals. However, Minina et al.^20^ revealed that FGFR3 signaling can accelerate the hypertrophic differentiation of chondrocytes in cultured limbs.^195^ It has also been reported that FGFR3 promotes the terminal hypertrophic differentiation of chondrocytes partially through MAPK.^20,205^ Activation of endogenous FGFR3 by FGF2 stimulation leads to reversible premature senescence of RCS cells.^206^ FGFR3 inhibits the synthesis of chondrocyte ECM such as aggrecan and collagen 2^199,207^ and promotes the degradation of ECM via stimulation of several MMPs, including MMPs 3, 9, 10, and 13 in chondrocytes, as a negative regulator of ECM.^208^ FGFR3 signaling is involved in macroautophagy of growth plate chondrocytes, which is important for the postnatal skeleton development.^209,210^ Recently, it was found that activated mutations of FGFR3 result in long bone defects potentially due to the dysfunction of primary cilia, including shortened length, reduced IFT20 trafficking, and aberrant HH signaling,^211,212^ suggesting that FGFs/FGFR3 may be involved in the function of primary cilia. Furthermore, FGFR3 directly and indirectly regulates the osteogenesis process. Mice carrying FGFR3 P244R mutation display thinning cortical bone and decreased bone mineral density in long bones.^213^ Our group found that FGFR3 can stimulate the osteogenic differentiation of BMSCs.^115^ Mugniery et al.^214^ revealed that FGFR3 from disorganized growth plate has a direct effect and an indirect effect on osteoblasts. Activation of FGFR3 in chondrocytes leads to premature closure of synchondrosis with enhanced osteoblastic differentiation through upregulation of the *BMPs* messenger RNA (mRNA) expression and downregulation of BMP antagonist.^215^ Consistently, *FGFR3* deficiency in chondrocytes promotes osteogenesis by stimulating differentiation and mineralization of osteoblasts through upregulation of *IHH*, *BMP2*, *BMP4*, *BMP7*, *WNT4*, and *TGF-β1*, and downregulation of *NOGGIN* expression.^216^ Both FGFR3 deficiency and constitutively activation lead to osteopenia and perturbed bone mineralization accompanied with changed osteoclastic activity,^115,217^ while FGFR3 has a direct positive effect on osteoclastic bone resorption.^218^

In general, FGFR1-3 all play critical roles in both chondrogenesis and osteogenesis, but FGFR3 is relatively more important in chondrogenesis.

### The role of FGF signaling in skeleton repair

Accumulating evidences have supported the crucial roles of FGFs/FGFRs in the injury repair of skeleton, including both cartilage and bone.

#### Endogenous FGF signaling in skeleton injury repair

##### Injury and degeneration of cartilage

Cartilage is an essential part of the skeleton. Growth plate is critical for the growth of long bone, while the articular cartilage provides smooth and low-friction interaction between the bones of joints.

Growth plate is fragile in growing skeleton. Given the role of FGF signaling in growth plate, it may play potential role in growth plate injuries. However, the roles of FGF signaling in growth plate injuries and healing is largely unknown. In young rat growth plate injury model, FGF2 is expressed in fibrogenic response phase and osteogenic stage coinciding with mesenchymal cell infiltration and bony bridge formation, suggesting the possible involvement of FGF2 in the repair of injured growth plates.^219^ In addition, FGF2 is involved in the regulatory role of tumor necrosis factor-α (TNF-α) in injured growth plates^220^ and contributes to the pathogenesis of osteoradionecrosis, osteopenia, and growth arrest.^221^

OA is a degenerative disease affecting mainly the articular cartilage. Human adult articular chondrocytes express FGFR1-4 with evident higher levels of FGFR1 and FGFR3, while the expression levels of FGFs/FGFRs were altered in the articular cartilage of OA patients.^222^ In human osteoarthritic chondrocytes, FGFR1 expression is increased with a concomitant suppression of FGFR3 expression.^223^ In murine models, disruption of FGFR1 in adult articular cartilage can delay the cartilage degeneration progression with downregulation of MMP13.^224^ ACH individuals resulting from FGFR3 GOF mutation exhibit a lower incidence of OA.^225^ Consistent with this, we revealed that FGFR3 delays OA progression in the knee joints and temporomandibular joints partially through downregulation of *IHH* in both spontaneous and surgically induced OA models in mice.^226,227^ Recently, we revealed that FGFR3 deficiency enhances the chemotaxis of macrophages via upregulating CXCR7, exacerbating the destruction of synovial joints.^228^ Both FGFs 1 and 2 are associated with radiographic phenotypes of knee OA at early phase.^229^ FGF1 is considered as a catabolic factor through down-regulating of CCN2 by interaction and enhancing the degradation of cartilaginous ECM by MMP13.^230^ FGF2 has both beneficial and deleterious effects on articular cartilage. In human articular chondrocytes, FGF2 can accelerate matrix degradation via a neuro-endocrine pathway^159^ and stimulation of ADAMTS5 expression through upregulating the transcription of *c-FOS/AP1* and *CBFA1*.^231^ On the contrary, FGF2 can promote the expression of *TIMP1* (tissue inhibitor of metalloproteinases 1) and suppress interleukin-1 (IL-1)-induced aggrecanase activity.^232,233^ Ablation of full-length FGF2 in mice accelerates the development of spontaneous and surgically induced OA.^155^ Deletion of LMW FGF2 isoform can accelerate murine OA, while loss of HMW FGF2 isoforms plays a protective role.^234^ Elevated FGF23 is involved in the role of HMW FGF2 in OA development by modulating Wnt/β-catenin signaling.^235^ FGF8 promotes the degradation of cartilage, leading to exacerbation of OA through enhancing the production of protease MMP3 and prostaglandin E2 produced by the injured synovium.^236^ We revealed that the expression of FGF9 is decreased with aging.^237^ Ellsworth et al.^238^ showed that FGF18 can act as an anabolic factor in cultured articular chondrocytes through stimulating collagen 2, proteoglycan accumulation, and chondrocyte proliferation.

##### Bone regeneration

Multiple studies have demonstrated that FGFs and FGFRs recapitulate their expression pattern in skeleton development during fracture healing process. In rat closed femoral fracture model, FGFR1 and FGFR2 have similar expression pattern; they are expressed in inflammatory cells, periosteal cells, chondrocytes, osteoblasts, and osteoclasts in fracture callus during both endochondral and intramembranous bone formation processes.^239,240^ The expression of FGFR3 is existed in mesenchymal cells, prehypertrophic, and hypertrophic chondrocytes in the fracture callus at a relative later stage.^240–242^ In mouse long bone fracture model, *FGFs* 1, 2, 5, 6, 9, and 16-18 are expressed throughout the healing process:^243^ *FGFs* 1, 2, and 5 are mainly expressed at inflammatory stage; *FGFs* 16 and 18 peak at endochondral bone formation phase; FGF2, 9, 16, and 18 are highly expressed, while FGF1 and 17 show peak expression at the bony callus formation and remodeling stage. FGF1 expression is increased during the formation of a cartilaginous callus in fracture,^244^ especially in fibroblast-like mesenchymal cells.^245^ In rat femoral distraction osteogenesis model, the expression of FGF2 was detected in fibrous mesenchymal cells, immature osteoblastic-like cells, and the periosteum adjacent to the areas of chondroid tissues.^246^

Skeletal phenotypes in mice with genetically modifying FGFs/FGFRs and the expression patterns of FGFs/FGFRs during fracture healing indicate the indispensable function of FGF signaling in bone regeneration. The SNPs of *FGFR1* are associated with fracture nonunion.^247^ We found that mice with FGFR2 GOF mutation (P253R) have enhanced bone formation induced by mechanical ablation of long bone marrow via upregulation of Wnt/β-catenin signaling.^248^ Our group using murine tibia fracture model reveal that FGFR3 plays a negative role in bone repairing through its regulation of both chondrogenesis and osteogenesis.^242,249,250^ In addition, FGFR3 inhibits the remodeling of injured tissue after cortical injury through downregulation of osteoclastic resorption.^218^ FGF1 may promote bone repair by inhibiting adipogenic differentiation and increasing the number of osteoblasts in the inflammatory environment.^251,252^ Using transgenic mice, Hurley’s group proved that LMW FGF2 accelerates the tibia fracture healing process through promoting chondrocyte and osteoblast differentiation and vascular invasion, and enhances the calvaria defect healing through canonical Wnt signaling.^253,254^ There is a strong positive association between plasma FGF21 levels and BMD in healthy women,^255^ although FGF21 promotes bone loss in mice.^256^ The serum FGF23 level may be a predictor of reduction of trabecular parameter and an indicator of nonunion.^257–259^

#### Application of FGF signaling modulators in skeleton repairment

##### Degeneration and injury of cartilage

FGFR1 promotes, while FGFR3 suppresses OA pathogenesis, suggesting that antagonists or neutralizing antibodies of FGFR1, and agonists or FGFs with high binding affinity for FGFR3, could be valuable therapeutics for OA. We revealed that pharmacologically antagonizing FGFR1 can alleviate OA progression in surgically induced mouse OA model and the osteoarthritic phenotype of cultured cartilage explants.^260,261^ As a high-affinity FGF ligand for FGFR3, exogenous FGF9 can attenuate cartilage degradation while aggravate osteophyte formation in murine post-traumatic OA model.^237^ In animal experiments, FGF18 has been repeatedly shown to have beneficial effects on OA and improve the healing of cartilage.^262–264^ To date, recombinant human FGF18 (rhFGF18) (trade name sprifermin) is the only FGF-based drug in clinical trials for OA. Clinic trial data show that intraarticular application of FGF18 can increase cartilage thickness and reduce cartilage loss without discernible local or systemic safety concerns.^265–268^ Exogenous FGF2 can enhance the repair of articular cartilage defect in vivo.^153,269,270^ FGF2 has also been used in combination with mesenchymal stem/progenitor cells to improve epiphyses repair.^271,272^ Due to the anabolic effect of FGF8 in the degradation of cartilage ECM, neutralizing antibody against FGF8 can partially alleviate the OA progression.^236^

##### Bone regeneration

Compared with the intervention of FGFRs, modulations of FGF signaling by ligands are closer to the clinical application. At present, more studies have been conducted on the application of exogenous FGFs in bone defect conditions.^273,274^

FGF1 in a sponge carrier has shown efficacy for bone regeneration as evidenced by more volume of new bone formation in rat critically sized cranial defect model.^275^ FGF1 with the fibrin carrier can promote bone regeneration of critically sized radial defect in rabbits.^276^ Kawaguchi et al.^277^ revealed that FGF2 in gelatin hydrogel could accelerate radiographic bone union of a surgical osteotomy in a dose-dependent manner, and promote tibial-shaft fracture repair with a safety profile in humans.^278^ FGF2 promotes the repair of bone injury mainly via inducing angiogenesis and enhancing the proliferation ability of osteoblastic lineage. However, the effect of FGF2 on bone formation in vivo is biphasic, with high-dose FGF2 having no stimulatory effect or inhibitory function. Sakano et al.^279^ found that injection of FGF2 (1 μg) markedly reduced the size of bone, and FGF2 completely inhibited ossification at a dose of 10 μg, during heterotopic bone formation induced by bone matrix powder implanted in murine hamstring muscles, indicating the inhibitory effect of FGF2 at a high dose on bone formation in vivo. Similar results have also acquired in a murine model putting collagen mini-pellet containing FGF2 into subperiosteal pouch, and in transosseous rat mandibular defects.^280^ Local delivery of FGF7 can enhance bone formation in rat mandible defects with enhanced osteogenesis and chemoattraction.^281^ Calvaria defects in either *FGF9* or *FGF18* haploinsufficiency mice showed impaired healing, which could be rescued by exogenous FGF ligands. FGF9-soaked collagen sponge causes sufficient bone regeneration in 2-mm diameter calvaria bone defects at postnatal day 7.^282^ Deletion of one *FGF18* allele can markedly reduce long bone regeneration with dramatic impairment of neovascularization, osteoclast recruitment, and bone remodeling, and treatment with FGF18 protein rescued the disturbed healing capacity.^283^ FGF18 application together with BMP2 can stabilize BMP2-dependent bone regeneration of 3-mm diameter critical-sized bone defects in mouse calvarium.^284^ Kang et al.^285^ established FGF2-FGF18-loaded fiber scaffolds to release FGF2 and FGF18 in a sequential manner, and found that it is effective for bone regeneration in rat calvarium defect model.

Our knowledge of the complicated roles and mechanisms of FGF signaling in bone regeneration is limited. The precise role of individual FGFs and FGFRs in individual cell lineage at different stages during fracture healing and bone regeneration, the application dose, timing and duration of FGFs, and its combination with other bone-modulating signaling molecules, novel vectors and protein delivery systems, need to be further explored to effectively promote bone regeneration and achieve better clinical applications.

## FGF signaling in lung development and diseases

The mammalian lung is derived through a series of epithelial branching events, leading to a complex branched airways and blood vessels, which eventually form a fully functioning air exchange organ. Lung development can be morphologically divided into several stages that correspond to key developmental transitions: the embryonic, pseudoglandular, canalicular, saccular, and alveolar stages.^286^ In chronological order, these stages involve endoderm induction, anterior-posterior and dorsal-ventral patterning, lung specification, lung budding, branching morphogenesis, and finally maturation.

### Expressions of FGF ligands and receptors in the lung

The expressions of FGF ligands and receptors have been found during lung development. Using in situ hybridization and RNA-sequencing, Danopoulos et al.^287^ assessed the expressions and distribution of FGF ligands in the cultured human fetal lung. It is demonstrated that the expression of FGF7 is in both the epithelium and mesenchyme; FGF9 is mainly expressed in the distal epithelium, while FGF10 is diffusely expressed throughout the parenchyma, and some expression of FGF10 is found in the smooth muscle cells (SMCs). FGFR2 is highly expressed in proximal, distal epithelial cells, and SMCs. FGFR3 is mostly expressed in the epithelial cells, and expressed lower in the mesenchyme, while FGFR4 is highly expressed in the mesenchyme and distal epithelium. The expressions of FGF ligands and FGFRs (FGFR1-4) also have been reported in the developing rodent lung.^288,289^

### Roles of FGF/FGFR signaling during lung development

FGF/FGFR signaling is essential for lung development. FGF1 stimulates lung epithelial cell proliferation and airway bud formation, and FGF7 causes cell proliferation in vitro inducing the formation of cysts from epithelia.^290^ Transgenic mice overexpressing FGF7 exhibit lung malformation.^291^ During the early phase of lung development, FGF9 controls epithelial branching and mesenchymal proliferation.^292^ Deletion or overexpression of FGF9 results in branching defects in mice with disturbance of the HH and Wnt/β-catenin pathway and the expressions of *FGF10* and *BMP4*.^293–295^ *FGF10* expression is drastically decreased in *FGF9*-deficient lungs from E14.5 onwards,^296^ and in FGF9-overexpressing lung, *BMP4* expression is increasingly expressed in the proximal and distal airway epithelium, whereas *FGF10* expression is upregulated locally in the distal mesenchyme.^293^ Deletion of *FGF10* results in complete distal lung agenesis.^297,298^ In cultured human fetal lung both FGF7 and FGF10 can induce liquid secretion and enlargement in distal tips.^299,300^ Using in vitro organoid cultures from the distal tip epithelium of human embryonic lung at pseudoglandular stage, Nikolic et al.^301^ have revealed that FGF10 is not required for the initial establishment of SOX2^+^/SOX9^+^ progenitors and for human lung branching. A recent study shows that foregut spheroids treated with high levels of FGF10 and 1% fetal bovine serum can form human lung organoids containing airway-like structures, mesenchymal cells, and alveolar epithelial cell type I and type II markers.^302^ FGF18 plays a role in lung alveolar development during late embryonic lung development. *FGF18* knockout mice show narrow alveolar space, thick interstitial mesenchymal compartments, and more embedded capillaries.^303^ Blocking the function of FGFR2 by a dominant-negative mutation results in blocked airway branching and epithelial differentiation.^304^ Mice deficient in both FGFR3 and FGFR4 show failure of alveogenesis, but deletion of either receptor alone does not disrupt lung development.^305,306^ A recent in vivo study demonstrated that FGFR3 and FGFR4 in mesenchymal cells have a function to control the organization of postnatal alveolar elastin, thereby driving the formation of alveolar septa for increasing the gas-exchange surface.^289^

### Roles of FGF/FGFR signaling in lung diseases

#### SNPs and mutations of FGFs/FGFRs in human lung diseases

Genetic analysis has found that SNPs in *FGFs* are associated with various types of lung diseases. SNPs in *FGF10* may be associated with susceptibility to chronic obstructive pulmonary disease (COPD).^307^ *FGF10* SNPs are also associated with airway branch variants.^308^ SNPs in *FGF3*, *FGF7*, and *FGFR4* are associated with respiratory distress syndrome (RDS). *FGFR4* (rs1966265) is also associated with bronchopulmonary dysplasia (BPD), the common chronic lung disease of premature birth.^309^ Besides, mutations in *FGFs* and *FGFRs* also have been found in human lung diseases. Mutations in *FGF10*, *FGFR2*, or *FGFR3* have been identified in LADD (lacrimo-auriculo-dento-digital) patients.^124,310^ Rare *FGF10* mutations have been identified in lethal pulmonary hypoplasia.^311^ Defects in the formation of tracheal cartilaginous ring resulting in mortality, resulting from respiratory distress, have been reported in Crouzon, AS, and PS caused by activating mutations of *FGFR2*.^312–314^ Homozygous loss-of-function mutation (R255Q) of FGFR2 contributes to ectrodactyly and pulmonary acinar dysplasia.^315^ All these findings suggest the crucial roles of FGF signaling in lung diseases.

#### Abnormal expressions of FGFs/FGFRs in lung diseases

In human fetal congenital cystic adenomatoid malformation, the epithelial FGF9 expression is 4-fold higher than that of normal fetal lung, whereas FGF10 and FGFR2 gene expressions have no change in the lung mesenchyme.^316^ Reduced FGF10 expression has been shown in BPD.^317^ FGF18 expression is decreased in hypoplastic lungs from patients harboring congenital diaphragmatic hernia (CDH).^318^ Plasma FGF23 levels is significantly elevated in COPD patients.^312^ FGF1/FGFR signaling is aberrantly increased in idiopathic pulmonary fibrosis (IPF) and may lead to the pathogenesis of lung fibrosis by promoting fibroblast migration via increased MAPK signaling.^319^

#### Regulation of FGF/FGFR signaling in lung diseases using in vivo and in vitro models

Studies in rodent models and in vitro lung cells have further implicated the roles of FGF signaling pathway in lung diseases. In lung of CDH rat, FGF7 and FGF10 gene expressions are decreased significantly compared with controls.^320^ Studies using rat doxorubicin-induced EA-TEF (esophageal atresia-tracheoesophageal fistula) model have found that disturbed FGF10/CTSH signaling is associated with impaired airway branching and consequent impairment of epithelial cells in the lung.^321^ BPD model established by exposing newborn mice to sublethal hyperoxia shows decreased expressions of *FGFR3* and *FGFR4*.^322^ Klotho knockout mice show COPD and airway inflammation with elevated FGFR4 in the lung, whereas airway inflammation was attenuated in mice with overexpression of klotho.^312^ FGF9 and FGF18 promote survival and migration of human lung fibroblasts from patients with IPF, and inhibit myofibroblast differentiation of human lung fibroblasts from patients with IPF.^323^ Recent studies have demonstrated that alveolar type 2 stem cells are maintained by FGF10-FGFR2B signaling. Loss of FGF10-FGFR2B signaling in bronchial epithelial cells leads to impaired generation of both neo-basal cells and alveolar epithelial cells after bleomycin injury, which can cause IPF.^324^ Deletion of FGFRs (FGFR1, 2, and 3) in lung mesenchyme decreases pulmonary fibrosis development in response to bleomycin.^325^ FGF7 and FGF10 can improve the lung repair and increase the epithelial survival after injury through FGFR2b signaling in rodents. FGF10 can also increase lung-resident mesenchymal stem cells and reduce the inflammatory response after acute lung injury (ALI).^326^ FGF10 has preventive roles in alveolar repair and resolution in ALI or acute RDS.^327^

### FGF/FGFR signaling as a target for the therapies of lung diseases

FGF/FGFR signaling represents a privileged target for the therapeutic approach. Therapeutics targeting FGF signaling pathways are largely classified into “pro-FGF signaling” and “anti-FGF signaling” therapeutics. Recombinant FGFs or FGF analogs have been developed as pro-FGF signaling therapeutics to improve the beneficial effects of FGF signaling. On the other hand, tyrosine kinase inhibitors (TKIs), anti-FGFR antibodies or peptides, and FGF traps have been found as approaches aimed to block FGF signaling.^328^ A TKI, Nintedanib, which targets FGFRs 1-3, PDGF receptors α/β, and VEGF receptors 1-3, has been approved in the USA and the EU to treat IPF.^329^ Recent studies found that FGF1 may have preventative and therapeutic effects on transforming growth factor-β1 (TGF-β1)-induced pulmonary fibrosis through inducing AEC proliferation, inhibiting myofibroblast differentiation, regulating TGF-β1 signaling, and FGFR1 expression. Thus, modulating FGF1 signaling may be a potential therapeutic strategy for the treatment of pulmonary fibrosis.^330^ Considering that FGF2 acts as an angiogenic mediator involved in various lung disorders such as COPD, pulmonary fibrosis, pulmonary hypertension, asthma, and lung cancer, FGF2 could also be an crucial target for the treatment of these lung disorders.^331^ FGF7 stimulates proliferation of lung epithelial cells and has been considered as a potential therapy for lung injury.^332^ FGF9 is a strong candidate contributing to the progression of IPF, which makes it a potential target for the therapies of IPF.^323^ Because of its important roles in lung development and diseases, FGF10 becomes an intriguing target for preventing and treating lung diseases.

However, FGF family is comprised of various ligands and receptors with multiple effects on different cell types in the lung, limiting the potential therapeutic efficacy. For instance, in contrast to its anti-fibrotic effect in TGF-β1-induced lung fibrosis, FGF1 and FGFR1-4 are also expressed increasingly in IPF lungs, and FGF1 treatment led to decreased collagen production and increased apoptosis of IPF-derived lung fibroblasts, suggesting that FGF1 may lead to the pathogenesis of lung fibrosis.^319^ Recent studies reported that FGF9 and FGF18 decreased normal fibroblast apoptosis, but had no effect on fibroblasts from IPF patients. FGF9, but not FGF18, decreased basal and TGF-β1-mediated expression of collagen and myofibroblast differentiation of fibroblasts.^323^ All these studies suggest that individual members of FGF family may exert variable effects, depending on the responding cells and the involvement of other signalings. Thus, investigation of specific roles of distinct FGF ligands and receptors in different types of lung cells will help to target differential pathways with precision and optimize the efficacy of future therapies for patients with lung diseases.

## FGF signaling in urinary system development and diseases

### Expression pattern of FGFs /FGFRs in kidney development

The metanephric kidney develops from nephrogenic cord and Wolffian (nephric) duct, which then generate ureteric bud (UB) and the metanephric mesenchyme (MM), respectively.^333^ FGFR1-4 and FGFs are highly expressed in mammalian embryonic kidney and lower urinary tract and play critical roles in the development of kidney. Although all FGFRs were detected in embryonic kidneys, FGFR3 or FGFR4 global knockout mice does not show significant structural defects of the kidney or bladder,^198,306^ which indicates that FGFR1, FGFR2, and FGFRL1 play more necessary roles in kidney development. FGFR1 is mainly expressed in MM lineages (early MM, developing into nephrons starting with vesicles and cap mesenchyme), the ureteric lineage, and renal cortical stroma.^334–337^ FGFR2 is mainly present in the Wolffian duct, the tips and trunks of UB, and differentiating nephrons, but has fewer expressions in early MM and stromal mesenchyme adjacent to the Wolffian duct.^338^ FGFRL1 is located in renal vesicles.^339^ The expressions of FGF 1, 2, 7, 8, 9, 10, 12, and 20 during kidney development have been reported.^338^ FGF2 can be secreted by ureteric tips. FGF1, 7, and 10 are expressed in renal stroma. FGF8 is mainly observed in the renal vesicle. FGF9 mostly locates in the UB as well as in the cap mesenchyme. FGF12 only presents in the UB. FGF20 is detected in nephron progenitors.

### FGFs/FGFRs in urinary system development

#### FGFs/FGFRs in nephron development

Early researches in rodents and *Xenopus laevi*s explants have found that exogenous FGF2 can maintain the sustained mesenchymal tissue growth and in some conditions induce formation of epithelial nephrons.^340–342^ More definitive evidences indicate the essential roles of FGF signaling in nephron formation. Deletion of *FGF8* with either *Pax3Cre*^343^ (in the MM) or *brachyury* (T) *Cre* (in mesodermal) line^344^ results in small kidneys with a complete block in nephron formation after the epithelial vesicle stage. Like the conditional *FGF8* knockouts, global deletion of *FGFRL1* also leads to blockade of nephron differentiation.^339^ These data indicate that FGFRL1 might be the candidate FGFR that binds to and mediates the effects of *Fgf8* in the nephron lineages.

FGF signaling also has positive effects on the maintenance of nephron progenitors. Among the growth factors known to have expression in embryonic kidney, FGF1, 2, 9, and 20 were found to promote proliferation of nephron progenitors in vitro.^345^ Global knockout of *FGF9* and *FGF20* alone or together led to nephron progenitor apoptosis and subsequent renal agenesis.^346^ Exogenous FGF9 or FGF20 is sufficient to maintain the stemness of MM or sorted nephron progenitors in vitro.^346^ However, *FGF1* knockout mice, alone and in combination with *FGF2* knockout, have no nephron progenitor defects,^347^ and *FGF2*-null mice^348^ have no renal defects. Mice with double knockout of *FGFR1* and *FGFR2* in *Pax3*-positive cells display severe defect of MM, while mice with either *FGFR1* or *FGFR2* deficiency have well-developed kidneys.^335^ These results indicate that FGFR1 and FGFR2 may have a redundant role in establishing and sustaining early MM. Conditional deletion of *FGFR1* and *FGFR2* with *Six2Cre* (in nephron progenitors) reduces Six2-positive nephron progenitors leading to severe renal cystic dysplasia.^349^ FRS2α is the main driver of FGFR signaling through ectopically activating notch signaling in nephron progenitors.^349^ Double mutation mice, carrying the point mutation in the FRS2α binding site of *FGFR2* and conditional deletion of *FGFR1* with *Pax3Cre*, show nephron progenitor depletion at later stages of development.^350^ Considering the similarity of the phenotypes in knockout mice, FGF9 and FGF20 are the likely ligands for FGFR/FRS2α in nephron development.

#### FGFs/FGFRs in ureteric branching and induction

FGF7 and FGF10 bind to FGFR2 and regulate the growth and branching morphogenesis of the collecting duct system. *FGF7*-null mice show marked reduction in developing ureteric bud and mature collecting system with secondary loss of nephrons.^351^ Meanwhile, FGF7 administration could augment ureteric bud growth and increase the number of nephrons in vitro.^351^ *FGF10*-null mice also have smaller kidneys with fewer collecting ducts.^352^ FGF7 and FGF10 activate the b isoform of FGFR2. Consistently, mice deficient for *FGFR2*-IIIb have dysgenesis of the kidney similar to that observed in *FGF7*- and *FGF10*-null mice.^353^

Recent studies further investigated the role of FGFR1 and FGFR2 in renal development using conditional knockout mice, since global deficiency of *FGFR1* or *FGFR2* leads to embryonic lethality prior to kidney development. Conditional loss of *FGFR2* in the Wolffian duct and its derivatives, including the ureteric bud using *Hoxb7Cre*, leads to renal hypoplasia, such as small ampullary, few ureteric branches, and thin trunks.^336,354^ Furthermore, neither knockout *FGFR1* alone nor double knockout of *FGFR1* and *FGFR2* with *Hoxb7cre* led to additional abnormalities beyond single knockout of *FGFR2*.^336^ Global deletion of *FGFR3* or *FGFR4* in mice results in no obvious gross phenotype of kidney.^198,306^ These data together suggest that among four FGFRs, FGFR2 seems to be the most important one regulating ureteric bud branching morphogenesis and stromal mesenchyme patterning.

### FGF signaling in kidney diseases

#### FGF and human genetic kidney diseases

Some mutations in FGFs or FGFRs in humans are associated with structural kidney and lower urinary tract diseases. Activating mutations of FGFR1, FGFR2, and FGFR3 lead to PS, AS, or TD. Some of these patients also have unilateral renal aplasia, hydroureter, vesicoureteral reflux, renal hypoplasia, and/or cystic dysplasia.^355^ Patients with Kallman syndrome due to LOF mutations in FGFR1 have unilateral renal aplasia. Inactivating mutations of FGF20 was found to cause bilateral renal aplasia.^346^

##### FGF signaling in CKD

Some endocrine FGFs (FGF21, FGF23) play important roles in CKD.

##### FGF21

FGF21 binds to a complex of KLB and FGFR1c to induce catabolic metabolism. Increased serum FGF21 levels are detected in CKD patients as early as stage 2.^356^ Since FGF21 was reported to have anti-aging effects, increasing the levels of FGF21 might be useful for the longevity of CKD patients.^36^ However, increased FGF21 also has many side effects. High FGF21 level can induce growth retardation, which might be related to the growth hormone resistance in children with CKD.^357^ Overexpression of FGF21 leads to osteopenia and increased adipogenesis in bone marrow that may contribute to the progress of CKD-mineral and bone disorder (CKD-MBD).^256^ High FGF21 may also be involved in the neuropsychiatric symptoms in CKD patients. Overexpression of FGF21 in mice causes disturbed circadian rhythm that can be rescued by specific ablation of KLB in the suprachiasmatic nucleus.^358^ Some researchers speculate that the circadian rhythm disorder related with high FGF21 level may contribute to the blood pressure fluctuation in CKD patients.^36^ FGF21 also increases serum corticosterone concentration that has been found to cause depression.^359,360^ Both depression and high FGF21 are associated with high mortality in dialysis patients.^361,362^ In brief, FGF21-KLB axis could be a potential treatment target in CKD.

##### FGF23

FGF23 is secreted from bone tissue and binds to a complex of α-Klotho and FGFR1c, FGFR3c, or FGFR4 in kidney as a hormone to regulate systemic phosphate homeostasis and vitamin D metabolism.^363^ A secondary elevation of serum FGF23 levels is commonly detected in CKD patients that are partly due to decreased renal clearance.^364^ The increased FGF23 is beneficial for lowering serum phosphate level and reducing 1,25(OH)_2_D_3_, which further increases the PTH level. These disturbed hormones would lead to CKD-MBD, which causes abnormities of bone turnover, mineralization, bone volume, extraskeletal calcification, and increased mortality.^365^ Clinical studies indicate that elevated serum FGF23 concentrations can be used to predict kidney disease progression, especially in the early stages of diabetic nephropathy.^366,367^ However, neutralization of FGF23 with its antibody further enhances the increased serum phosphate level and vascular calcification that can cause increased risk of mortality.^368^ The direct role of elevated FGF23 in the cardiovascular events caused by CKD should be further studied. Furthermore, increased serum FGF23 level may be a beneficial compensatory response to maintain mineral homeostasis in early stage of CKD. FGF23 is not only a biomarker for the diagnosis and/or prognosis of CKD, but also a pathogenic factor for the progression of CKD and cardiovascular disease. Targeting the FGF23-Klotho endocrine axes might have therapeutic benefit for diseases of kidney in clinics.^36^ Whether blocking of FGF23 activities in patients with end-stage renal disease is an effective therapy to improve symptoms needs to be further studied.

Recently, FGF23 has been found to regulate immune system in CKD. Impaired immunological responses and susceptible to infections are common in CKD patients.^369,370^ Circulation FGF23 level is correlated with incidence of infections.^371^ Previous studies suggest that FGF23 might be intimately involved in inflammatory processes. FGF23 increases the number of macrophages and induces the expression of TNF-α in response to inoculation with *Escherichia coli* or *lipopolysaccharide* injection.^372^ The stimulation of TNF-α in M2 macrophages by FGF23 could be blocked by 1,25(OH)_2_D_3_.^373^ FGF23 inhibits arginase-1 expression in M2 macrophages.^373^ These studies suggest that FGF23 has pro-inflammatory functions. It was further reported that FGF23 prevented leukocyte recruitment and impaired host defense in CKD.^374^ FGF23-α-Klotho-FGFR2 axis plays a central role in this process by activating PKA and inhibiting Rap1 that will finally inactivate β2-integrin function.^374^ FGF23 could also facilitate the rolling of neutrophils.^374^

Fibrosis is a common feature of CKD, and involves leukocyte recruitment, angiogenesis, blood vessel leakage, and appearance of myofibroblasts. Secretion of FGF2^375^ and FGF23^376^ from podocytes, mesangial cells, interstitial mesenchymal cells, endothelia, or myofibroblasts was reported. FGF2 facilitates the trans-differentiation of tubular epithelial cells to mesenchymal cells, which accelerates the increase of matrix-producing cells.^375^ However, detailed mechanisms for the role FGF signaling in renal fibrosis remain to be explored.

#### FGF signaling in kidney injury and repair

Elevated FGF23 levels in the circulation and urine were reported in acute kidney injury (AKI) patients by numerous studies.^377–381^ Increased serum FGF23 level has been found to be an early marker of incident AKI. In three independent cardiac surgery cohorts, patients with AKI have higher levels of C-terminal FGF23 (cFGF23) than those who did not develop AKI as early as cardiopulmonary bypass ending.^377,378,382^ The predictive performance of cFGF23 was higher than other urinary injury biomarkers, including NAG (*n*-acetyl-*b*-(d)-glucosaminidase), KIM-1 (kidney injury molecule-1), and NGAL (neutrophil gelatinase-associated lipocalin) at the end of cardiopulmonary bypass.^377^ FGF23 is also thought to be a candidate prognostic marker for the adverse outcomes in AKI patients. Patients with the highest quartiles of cFGF23 and intact, biologically active protein (iFGF23) had a significantly increased risk of 60-day mortality than those having the lowest quartiles in two cohorts of critical illness involved AKI patients.^383^ Further study is required to clarify whether aberrant FGF23 contributes to the poor outcomes of AKI.

The mechanisms underlying the increased plasma FGF23 in AKI are not clear. Increased production of FGF23 in osteoblasts may be one of the major causes. Increased mRNA expressions of *FGF23* in the bone, bone marrow, and renal tissues are found in several AKI mouse models.^384–386^ This could be reversed by pretreatment with PD173074, an FGFR inhibitor, or blocking the erythropoietin receptor.^384,386^ These results indicate that the increased circulating erythropoietin and erythropoietin receptor activation are involved in the mechanisms leading to increased plasma FGF23 in AKI. Resection of the obstructed kidney had no effect on the increased circulating iFGF23 levels,^387^ excluding the possibility that production of FGF23 by the kidneys contributes to plasma FGF23.

Considering the relevance of FGF signaling in kidney development and diseases, there may be potential therapeutic strategies to regulate the process of renal development and diseases by manipulating FGF signaling. For example, recombinant FGF10 may be useful in alleviating ureteric branching defects in Fraser syndrome (FRAS1 mutations).^388^ The requirement for FGFR2 signaling in lower urinary tract mesenchyme^389^ suggests that FGF-related therapies could be used to repair the smooth muscle defects in the ureter or bladder. FGF7 expression levels are increased after chemically induced kidney injury in rats.^390^ Intravenous administration of recombinant truncated human FGF7 largely prevented cyclophosphamide-induced urothelial injury in rats,^391^ indicating that FGF7 could be a potential therapy for patients with bladder urothelial injury.

## FGF signaling in muscle and heart development and disease

### FGF signaling in the skeletal muscle

Adult skeletal muscle possesses remarkable regeneration capacity; it can be rapidly repaired after the damage caused by exercise, trauma, toxins, or diseases.^392^ Satellite cells (SCs), which reside beneath the basal lamina of muscle fibers, are considered as the stem cells in the skeletal muscle. Normally SCs are mitotically quiescent, but upon regeneration they are activated, and give rise to myogenic precursors.^392^ After several rounds of proliferation and differentiation, most of these myogenic precursors form new muscle fibers, while a small population of these cells returns to quiescent SCs.^392^

#### FGFs in the skeletal muscle

FGFs are essential for the self-renewal of SCs and are needed for skeletal muscle maintenance and regeneration. *FGF1*, *FGF2*, *FGF4*, and *FGF6* can be detected in SCs.^393,394^ *FGF1* and *FGF4* can be found in isolated myofiber cultures and in in vivo injured adult skeletal muscle tissue.^394,395^

##### FGF2

FGF2 is present in the extracellular matrix and basal lamina of skeletal muscles,^396^ and is produced by fibroblasts,^397^ myofibers,^398^ and SCs,^399^ while the relative contribution of FGF2 to these cells is difficult to distinguish. FGF2 has been used as a routine medium supplement in SC primary culture.^400,401^ Although SCs from young mice (3–6 months) do not need supplementation of FGF2 in the culture medium, SCs from geriatric mice (29–33 months) cannot proliferate without the addition of FGF2.^402^ FGF2 is considered as a mitogen for SCs; it triggers SC proliferation by repressing myogenesis.^403,404^ However, FGF2 is not able to stimulate cell division without serum.^405,406^ Recently, it is reported that excessive FGF2 removes age-associated proliferative inhibition of SCs.^407^ The upregulated expression of *FGF2* in aged muscle fibers and downregulated expression of *SPRY1* in aged SCs increase the FGF signaling under homeostatic conditions and break the quiescence of SCs, resulting in SC depletion and losing self-renewing capacity.^408^ SPRY1, an inhibitor of FGF signaling, is highly expressed in quiescent adult SCs in uninjured muscle,^409^ while muscle stem cell niche, the muscle fiber, expresses FGF2 under homeostatic conditions. Spry1 is needed for the maintenance of the endogenous adult Pax7-positive SCs in their native environment, but it is downregulated in proliferating myogenic progenitors in injured muscles.^410^ Overexpression of *SPRY1* in SCs or inhibition of FGFR1 signaling can prevent SC depletion. Thus, blockade of FGF2/FGFR1 signaling might be a new therapeutic method to recover the regeneration capacity of skeletal muscles during aging.^408^ The expression of FGF2 is found to be increased during the muscle regeneration,^398^ and exogenous FGF2 could promote muscle regeneration in dystrophic mice.^411^ However, this effect is wiped out in *FGF2*-null mice,^348^ and injection of FGF-blocking antibodies also inhibits the regeneration process.^412^

##### FGF6

FGF6 can be detected in both embryonic and adult skeletal muscle tissues,^413,414^ and isolated myofibers.^394^ In adult mice, FGF6 is secreted by fast-twitch fibers, and its expression is increased after skeletal muscle injury.^415^ FGF6 mainly performs its function through binding to FGFR4.^416^ Presently, the role of FGF6 in the skeletal muscle is controversial. Interbreeding of *FGF6*-deficient mutants with dystrophic mdx mice (a model for Duchenne muscular dystrophy) results in tremendous dystrophic changes in skeletal muscles, including degeneration of myotube, emergence of many mononuclear cells, and collagen deposition. MyoD mRNA is normally upregulated in mdx; however, it is not observed in double mutant mice.^415^ It is also reported that *FGF6*-deficient mice show regeneration defects with myotube degeneration and severe fibrosis.^415^ The numbers of Myo^+^Myogenin^+^ activated SCs are severely reduced in mutant mice after injury, and which is not caused by the decreased quiescent SCs, probably by the lack of activated SCs.^415^ However, another team declared that no skeletal muscle phenotype is found in *FGF6*-deficient mice, and FGF6 might not play an essential role in muscle regeneration or its function is compensated by other FGFs.^417^ Using *FGF6* global knockout mice and rescue experiments, Armand et al.^418^ found that FGF6 is participated in soleus regeneration of adult mice in a specific dose-dependent manner: FGF6 promotes the proliferation of the myogenic cells at high doses, while it regulates the differentiation of myogenic cells and muscle phenotype via a calcineurin signaling pathway at lower doses. Genetic deletion of *FGF2* and *FGF6* in mdx mice leads to much more severe dystrophic phenotypes in *FGF2/FGF6/MDX* triple-mutant mice than in mdx mice,^419^ which further supports that FGF6 plays an important role in muscle regeneration.

##### FGF15/19

Recently, FGF19 has been reported to have novel function in enlarging muscle fiber size, and in protecting the skeletal muscle from atrophy.^420^ Pharmacological dosage of FGF19 significantly increases human myotube size in vitro.^420^ Treatment of mice with FGF19 causes skeletal muscle hypertrophy, while genetic deletion of KLB eliminates the hypertrophic effect of FGF19 in mice.^420^ Both in vitro and in vivo, FGF19 stimulates the phosphorylation of ERK1/2 and the ribosomal protein S6 kinase (S6K1), which is an mTOR-dependent key regulator of muscle cell growth.^420^ Studies also found that FGF19 relieves the skeletal muscle wasting induced by glucocorticoid, obesity, or sarcopenia in mice. Therefore, FGF19 have the therapeutic potential for promotion of the skeletal muscle mass and treatment of muscle wasting.^420^

#### FGFRs in the skeletal muscle

Among the four FGFRs, SCs express high levels of FGFR1 and FGFR4, low levels of FGFR3, and little or no detectable FGFR2.^404,421^ However, studying the relative contributions of the FGFRs to SCs is rather difficult, because they usually activate multiple intracellular signaling pathways and their functions are often compensated by each other when inhibited by one of the FGFR.

##### FGFR1

FGFR1 is highly expressed in freshly isolated SCs and myogenic cultures, and it has been considered in the context of adult myogenesis.^394,422^ *FGFR1*-null mice cannot gastrulate.^423,424^ Myogenic-specific (MyoDCre-driven) ablation of *FGFR1* in mice seems to have no overt effect on the histology characteristics of muscle and the progress of muscle regeneration following cardiotoxin-induced injury.^404^ In contrast, SCs could not respond to the stimulation of FGF2 in isolated myofibers from *FGFR1*-ablated mice,^404^ which suggests that other FGFRs may compensate the function of FGFR1 during SC differentiation. FGFR1 downstream signals include both ERK regulating SC proliferation^425^ and p38α and p38β (p38α/β) MAPK pathways that is involved in the exit of SCs from quiescence,^426,427^ asymmetric division of SCs,^427^ and differentiation of SCs in vivo.^427^ Recently, it is reported that SCs from aged mice autonomously lose their self-renewal ability due to alterations in FGFR1, p38α, and p38β MAPK signaling.^428^ Ectopic activation of phospho-FGFR1 partially rescues their age-associated self-renew ability with asymmetric localization of phospho-p38α/β MAPK in dividing SCs.^428^ These results highlight an age-associated deregulation of homeostatic network of SCs and hints a therapeutic potential for the treatment of muscle wasting.

##### FGFR4

FGFR4 is expressed in intact myofibers, muscle connective tissue, isolated proliferating and differentiating SCs in culture.^394^ FGFR4 plays a role in cell fate determination during embryonic muscle development.^429^ However, FGFR4-null mice are healthy and fertile with no evident muscle defects, which hints that FGFR4 is dispensable during embryonic development.^306^

### FGF signaling in the heart

Unlike other tissues and organs such as muscle, blood, and liver, the mammalian heart possesses very limited regenerative capacity. Mammalian cardiomyocytes could robustly proliferate in the second heart field during early organogenesis.^430^ However, recent lineage tracing studies dubbed c-Kit-positive cardiac stem cells (CSCs), which had no cardiogenic activity and could not support heart repair in adulthood.^431–434^ Instead, the injured myocardium develops scar and fibrosis.^435^ Thus, researchers have been tempted to uncover the mechanisms of the cardiogenesis and regeneration, which may make it possible to stimulate and manipulate the regenerative potential of heart. FGF signaling pathways, especially FGFs, have been shown to be highly involved in the cardiac development, diseases, and repair.

#### FGF1

FGF1 together with TNF-related weak inducer of apoptosis (TWEAK), by binding to FGFR1, could induce cardiomyocyte cycle re-entry.^436^ This effect can be blocked by inhibiting the TNF receptor superfamily member FGF-inducible molecule 14. TWEAK induces the activation of cardiomyocyte cycle, which can be inhibited by blocking FGFR1 signaling.^436^ Co-stimulation experiments showed that FGF1 and TWEAK could regulate the cardiomyocyte cycle induction via PI3K/AKT signaling.^436^ It is also reported that the treatment of FGF1 stimulation and p38 inhibition have protective effect on ischemic heart disease by inhibiting cardiomyocyte apoptosis.^437,438^ In vitro postnatal mammalian cardiomyocytes can proliferate under the FGF1 stimulation and p38 MAPK (p38) inhibition,^439^ and the combination treatment also increases cardiomyocyte mitosis after acute myocardial injury in 8–10-week-old rats. Four weeks after injury, the treatment reduces heart scarring, wall thinning, and markedly rescues cardiac function.^439^ However, cardiac-specific overexpression of *FGF1* only delays the formation of myocardial infarct, but has no significant effect on maximal infarct size.^440^ In contrast, inhibition of p38 fails to rescue heart function despite increased cardiomyocyte mitosis. These results imply that FGF1 might promote the survival of newly generated cardiomyocytes through the enhancement of angiogenesis.^439^ Even so, the combination of FGF1 stimulation and p38 inhibition may have therapeutic effect by improving human cardiac regeneration.^435^

#### FGF2

FGF2 is widely expressed in murine heart. In FGF2 transgenic mice, the hearts exhibit exacerbated cardiac hypertrophy assessed by myocyte cross-sectional area and heart weight-to-body weight ratios, which is eliminated in the presence of ERK inhibitor, but not p38 pathway inhibitor.^441^ In contrast, the chronic elevation of blood pressure, fibrosis, and hypertrophy induced by two-kidney one-clip can be attenuated in *FGF2* knockout mice.^442^ Isoproterenol-induced and myocardial infarct-induced cardiac fibrosis and hypertrophy can also be attenuated in *FGF2* knockout mice.^441,443^ Besides, FGF2 is a cardio-protector in myocardial infarction models and ischemia/reperfusion (I/R) injury.^444^ The expression of FGF2 is shown to be upregulated after a cardiac injury.^445^ FGF2 inhibits the autophagy and increased the clearance of ubiquitinated protein through PI3K/AKT/mTOR signaling in mouse myocardial I/R injury model.^446^ FGF2 also suppresses endoplasmic stress and mitochondrial dysfunction through PI3K/AKT and RAS/MAPK signaling pathways.^446^ Therefore, FGF2 is being tried for treating ischemic conditions in several clinically relevant trials.^447–449^

#### FGF9

FGF9, expressed in the endocardium and epicardium, regulates cardiomyocyte proliferation during embryogenesis,^450^ and newborn *FGF9* knockout mice develop a dilated cardiomyopathy due to premature differentiation of cardiomyocytes.^450^ FGF9 is also shown to improve systolic function and heart failure mortality by stimulating the hypertrophy of non-infarcted left ventricular after myocardial infarction with increased microvessel density (MVD), reduced fetal gene expression, and interstitial fibrosis in myocardium-specific transgenic *FGF9* mice.^451^ However, FGF9 only stimulates the network formation and the proliferation of endothelial cells (ECs) without induction effects on myocardial hypertrophy in culture.^451^ It is reported that FGF9 can mediate the differentiation of monocytes to M2 macrophages; FGF9 treatment of an infarcted myocardium in diabetic mice increased anti-inflammatory cytokines and M2 macrophage differentiation, which resulted in reduced adverse remodeling and improved cardiac function.^452^ Therefore, FGF9 may have novel therapeutic potential for this type of myocardial infarction.

#### FGF10

FGF10 is found in the second heart field during early heart development,^430^ and also expressed in progenitors for the right ventricle and outflow tract.^453^ Neonatal mouse hearts possess the regenerative ability, but gradually lose this ability after postnatal day 7.^454^ FGF10 is reported to promote regional fetal cardiomyocyte proliferation and cell-cycle re-entry of adult cardiomyocytes, but has no effect on fibroblasts that is mediated by FOXO3/P27.^454^ In addition, *FGF10* deficiency mice display misplacement of the heart in the thoracic cavity with right ventricular hypoplasia due to reduced cardiomyocyte proliferation.^455^ In contrast, overexpression of *FGF10* in the myocardium of mice promotes cardiomyocyte proliferation after heart injury without the increase of epithelial-to-mesenchymal transition and fibrosis;^456^ thus, FGF10 may be a potential drug for cardiac repair.

## FGF signaling in angiogenesis, lymphangiogenesis, and related diseases

Angiogenesis or lymphangiogenesis is the process of vascular or lymphatic formation during physiological and pathological conditions, such as embryogenesis, trauma, inflammation, and tumor development. Since lymphatics can be derived from the sprouting of veins, lymphangiogenesis is considered to be associated with angiogenesis.^457^ FGF/FGFR signaling has been demonstrated to play important roles in angiogenesis and lymphangiogenesis.

### Expressions of FGFs/FGFRs during angiogenesis and lymphangiogenesis

FGFR1 is expressed in vascular ECs and *FGFR1* knockdown leads to upregulated FGFR3 expression in the endothelium.^458^ *FGFR2* was found expressed in murine aortic endothelium.^459^ ECs express the FGFR1IIIc, FGFR2IIIc, and FGFR3IIIc isoforms of FGFRs, but not the IIIb isoforms nor FGFR4, and vascular SMCs (VSMCs) express the similar isoforms of FGFRs; several FGFs are expressed in ECs (FGFs 1, 2, 5, 7, 8, 16, and 18) and VSMCs (FGF1, 2, 5, 8, 16, and 18). FGFR1 and FGFR3 are expressed in lymphatic ECs (LECs) during lymphangiogenesis as demonstrated by several studies,^458,460^ and they were reported to be critical for the lymphatic formation.

### FGF signaling in vascular and lymphatic formation

FGF signaling can influence the whole process of angiogenesis. Activation of FGFR1 or FGFR2 has been demonstrated to have a positive effect on vascular endothelial proliferation.^461^ One important step of angiogenesis is extracellular matrix degradation. Some FGFs, including FGF1, FGF2, and FGF4, promote the expressions of MMPs in ECs.^462^ FGF2 can stimulate shedding of MMP2 and MMP9 in cell surface membrane vesicles from ECs, which is able to stimulate the angiogenesis of ECs seeded in Matrigel.^463^ Another essential step of angiogenesis is endothelium migration. FGF1, FGF2, FGF8, and FGF10 were demonstrated to stimulate endothelium chemotaxis.^464^ The pro-chemotactic effect of FGF2 depends on activation of MAPK.^465^

The role of FGFR3 in lymphangiogenesis is controversial. It is revealed that *FGFR3* is a novel target gene of *PROX1*, which is essential for lymphatic development. Knockdown *FGFR3* by small interfering RNA (siRNA) inhibited LEC proliferation.^460^ Meanwhile, 9-*cis* retinoic acid (9-*cis*RA) was reported to activate FGF signaling and enhance lymphatic formation and regeneration by promoting the proliferation, migration, and tube formation of LECs.^466^ FGFR3 expression in LECs was upregulated after 9-*cis*RA treatment. 9-*cis*RA-induced LEC proliferation and migration were significantly inhibited by soluble FGFR3 recombinant protein as well as FGFR inhibitor PD173074.^466^ However, Yu et al.^458^ showed that FGFR3 alone is not enough to influence lymphangiogenesis. Vascular and lymphatic vessel defects were observed in *FGFR1/FGFR3* double mutant mice, but single knockout of *FGFR1* or *FGFR3* led to no abnormality in lymphatic front migration in embryonic mouse skin examined by whole-mount staining for VEGFR3 (vascular endothelial growth factor receptor 3) and PECAM1 (platelet and endothelial cell adhesion molecule-1). The controversial effects of FGFR3 on lymphatics may be due to its differential influence on LECs during embryonic phase or adulthood.

### FGF/FGFR-related diseases with abnormal angiogenesis and lymphangiogenesis

There are few clinical reports about the relationships between FGFs/FGFRs and diseases with abnormal angiogenesis and lymphangiogenesis. Some experimental results demonstrate that FGFs/FGFRs may play an essential role in diseases with abnormal vascular formation. Many tumor cell lines produce FGF2.^467^ Inhibition of FGFR1 by FGF2 antisense complementary DNAs (cDNAs) suppressed vascularization and growth of human melanomas in nude mice.^468^ Furthermore, FGF levels were correlated with intratumoral MVD, an important parameter for tumor progression.^469^ In some tumors like melanoma, FGF2 level has a strong correlation with MVD and clinical outcome of the patients.^469^ However, whether FGFs/FGFRs also influence tumor parenchyma needs to be further clarified. Inflammation is an important trigger for angiogenesis. It is revealed that monocytes, mononuclear phagocytes, and mast cells express FGF2.^470^ Inflammatory cytokines including IL-1β can stimulate FGF2 production in ECs.^471^ Inflammatory mediators might stimulate angiogenesis through increasing FGF signaling in endothelium. EC death can lead to increased FGF2 release. Hypoxia upregulates VEGF and FGF2 production and increases endothelial responsiveness to FGF2.^472^ The activity of FGF/FGFR signaling may be strongly associated with inflammation and influence angiogenesis at multiple levels.

### FGF signaling and EndMT

Endothelial-to-mesenchymal transition (EndMT) is the process through which ECs transform into mesenchymal cells. EndMT plays important roles in the pathogenesis of various human diseases, including cardiac fibrosis, atherosclerosis, and heterotopic ossification (HO).^473^ EndMT was first confirmed in animal models in which Tie1^+^ endothelials adopted cardiac fibroblast fate during cardiac fibrosis development.^474^ Further investigations found that Tie2^+^ vascular ECs contributed to HO formation in fibrodysplasia ossificans progressive and BMP4-induced HO mouse models.^475^ Currently, TGF-β1 signaling is regarded as the main inducer of EndMT.^473^ FGF signaling has recently been demonstrated to downregulate TGF-β signaling and inhibit EndMT. Basal FGF signaling maintains endothelial homeostasis through inhibiting the expressions of TGF-β, TGF-βR1, and SMAD2 via controlling the *let-7* microRNA (miRNA) levels.^476^ Meanwhile, in vitro and in vivo experiments showed that inflammatory cytokines, including interferon-γ, TNF-α, and IL-1β, decreased FGFR1 expression, leading to reduced FGF signaling activation in ECs.^476^ Another study reported that FGF2 can induce miRNA-20a expression, which represses TGF-β signaling in endothelium and inhibits EndMT.^477^ Therefore, it is plausible that FGF signaling downregulation by inflammatory cytokines contributes to vascular neointima formation and fibrosis driven by TGF-β-induced EndMT.

### Therapeutic modulation of angiogenesis and lymphangiogenesis

Therapeutic angiogenesis is a promising approach to the recovery of ischemic diseases. It was shown that intracoronary FGF2 administration preserved myocardial function by increasing vascularization.^478^ Some clinical trials demonstrated that FGF2 administration can improve the symptoms of patients with coronary artery disease or peripheral artery disease.^448,479^ In addition, inhibition of *FGF2/FGFR1* by antisense cDNAs blocked intratumoral angiogenesis and arrested the growth of human melanomas grown subcutaneously in nude mice.^468^

FGF-based angiogenic therapy has been shown to be a potential treatment for patients with ischemic diseases. However, many details including timing, dosage, application alone, or in combinations with other drugs and effective delivery approach need to be further clarified. There are few reports about the therapeutic modulation of lymphangiogenesis based on FGF signaling. 9-*cis*RA was reported to have a therapeutic effect on lymphatic regeneration and secondary lymphedema in experimental mouse models, which could be dependent on FGF signaling in LECs.^466^

## FGF signaling in inflammatory response

Inflammation is a complex adaptive response that can be induced by endogenous and exogenous substances/stimuli.^480^ Besides the recognition of inducers, inflammatory response includes multiple process such as the production of multiple inflammatory mediators, including inflammatory factors, chemokines, and vasoactive amines, which are released by immune cells like macrophages and mast cells.^480^ There are lots of studies reported that FGFs/FGFRs play important roles in the regulation of inflammationory response.

### FGFs in inflammation

#### FGF1 in inflammation

FGF1 can accentuate inflammatory response.^481^ Generally, FGF1 is highly expressed in the inflammatory cells and tissues. High levels of FGF1 can be found in multiple tissues of inflammatory arthritic joints, including bone, cartilage, synovium, ligament, and tendon.^482^ Besides, most T cells in synovial tissue in rheumatoid arthritis express FGFR1 for FGF1.^483^ FGF1 can enhance IL-2 production and activation of NF-κB in T cells.^483^ Rossini et al.^484^ found that both FGF1 and FGFR1 are expressed in filtrating lymphocytes and macrophages during the renal inflammation, and FGFR1 is highly expressed in tubules, suggesting that FGF1 might have both autocrine and paracrine functions. Hackshaw and Shi^485^ reported that FGF1 affects the calcium mobilization and increases the level of cytosolic calcium in macrophages. FGF1 causes ATP release from spinal astrocytes and opens gap junction channels after spinal cord injury, which may aggravate the inflammation in neurological disease and injury.^486,487^ Recently, Huang et al.^488^ engineered the FGF1 mutants (termed FGF1^ΔHBS^) with reduced ability to activate FGFR, and found that FGF1^ΔHBS^ inhibited inflammation and oxidative stress in CKD via activating PI3K/AKT and GSK-3β/Nrf2 signaling pathways, which inhibited the ASK1/JNK.^489^ The results suggest that FGF1 bears the responsibility of anti-inflammation, especially in certain chronic inflammatory diseases. Besides, FGF1 has the ability of anti-inflammation in diabetic nephropathy via inhibition of JNK (c-Jun N-terminal kinase) and NF-κB pathways.^490^ Thus, the effects of FGF1 on inflammation may vary from different diseases and conditions.

#### FGF2 in inflammation

FGF2 is also involved in several inflammation-related diseases such as multiple sclerosis and rheumatoid arthritis.^491^ Ectopic expression of FGF2 exacerbates inflammatory response and symptom of colitis and collagen-induced arthritis models.^492,493^ FGF2 contributes to the inflammation in articular cartilage during the process of OA.^494^ Besides, the level of FGF2 is increased during the whole blood inflammatory reaction induced by the artificial surface.^495^ During the infection of HIV, FGF2 shows a positive correlation with the number of CD4^+^ T cell.^496^ FGF2 induces the expression of RANKL via ERK1/2 activation in human bone marrow mesenchymal stromal cells, which suggests that FGF2 may play the osteoimmunological role during bone regeneration.^497^ Pawlowski et al.^498^ found that FGF2 is highly expressed in fibroblasts and adipocytes, and FGF2 may contribute to perpetuation of inflammation in the orbital tissue of Graves’ orbitopathy. FGF2 has close relationship with inflammatory response during angiogenesis such as activation of pro-inflammatory chemokines in ECs and engagement of monocyte/macrophage.^499^ FGF2 increases the concentrations of cellular IL-1β in human VSMCs.^500^ The above studies indicate that FGF2 has the function of pro-inflammation. However, exogenic FGF2 could attenuate inflammatory response such as the decreased expression of IL-1β in epileptogenesis-associated neuroinflammation.^501^ In addition, inhaling recombinant FGF2 decreases lung inflammation in asthma and COPD.^502,503^ Thus, targeting FGF2 is a potential method to alleviate certain inflammatory diseases such as neuroinflammation, asthma, and COPD.

#### FGF3/FGF21/FGF23 in inflammation

Unlike FGF1 and FGF2, there are few studies that reported the relationship between FGF3 and inflammation. The level of FGF3 in sinonasal tissues is significantly upregulated in acute allergic rhinitis and chronic sinonasal inflammation mouse models.^504,505^ However, FGF3 level in middle ears is significantly downregulated in mouse model for acute otitis media.^506^ Combining these results, we speculate that the role of FGF3 in inflammation may be distinct in the different tissues/organs.

FGF21 can be induced by inflammatory stimuli.^507–509^ FGF21 is associated with the suppression of cardiac, renal, and hepatic inflammation.^510–512^ FGF21 is thought to be one of the potential immunotherapy targets for cardiovascular inflammation and pancreatic fibrogenesis as it can alter the macrophage polarization states.^513,514^ Exogenous FGF21 was found to alleviate soakage of inflammatory cells in the lung potentially via elevation of IL-10.^515^ FGF21 inhibits macrophage migration and significantly reduces inflammatory factor expression in oxidized low-density lipoprotein-induced THP-1 macrophages.^516^ In addition, FGF21 can also repress inflammatory factors induced by insulin resistance.^517^ FGF21 has anti-inflammatory effect on preadipocytes via FRS2/ERK1/2 signaling pathway.^518^ Besides, FGF21 can suppress the production of IL-1β mediated by NLRP3 (NOD-, LRR- and pyrin domain-containing protein 3) inflammasome.^519^ In general, inflammation increases the expression of FGF21, which is an anti-inflammatory factor in many diseases.

The relationship between inflammation and FGF23 may be bidirectional.^520^ Lang et al.^521^ suggested that the increase of FGF23 induced by inflammatory signaling may amplify inflammation by suppressing the synthesis of the anti-inflammatory 1,25(OH)_2_D_3_ in inflammatory diseases. Besides, FGF23 can induce multiple inflammatory signaling pathways like TNF-α signaling. In addition, FGF23 activates calcineurin signaling by activating FGFR4 in hepatocytes, which causes the increased level of inflammatory cytokines in CKD.^522^ In summary, inflammatory response can induce the expression of FGF23 and FGF23 can act as a pro-inflammatory factor.

### FGFRs in inflammation

In addition to the FGFs, the receptors of FGFs also play important roles in inflammatory response. FGFR1 promotes inflammation via activating NF-κB signaling pathway in prostate cancer cells.^523^ However, FGF2/FGFR1 pathway has inhibitive effects on astrocyte-mediated neuroinflammation after infrasound exposure.^524^ In turn, there is a profound reduction in FGFR1 in human umbilical vein ECs treated by TNF-α and IL-1β, while other inflammatory cytokines such as IL-6 could not inhibit the expression of FGFR1.^476^ Besides, our group recently identified that FGFR3 deficiency promoted chemotaxis of macrophages via activation of NF-κB/CXCR7 signaling pathway, which reveals the negative role of FGFR3 in synovial inflammatory response.^228^ More studies about the roles of FGFRs in inflammation are needed in the future.

Inflammatory response is regulated by multiple factors in a variety of cellular behaviors.^525^ Targeting pro-inflammatory factors such as IL-6 and TNF-α has been shown to be an effective therapy for some inflammatory diseases, and therapeutic antibodies are also promising strategy to treat inflammatory diseases.^526,527^ From the above studies, we can conclude that FGF signaling has close relationships with inflammatory response, and whether it exerts a pro-inflammatory or an anti-inflammatory role mainly depends on the types of FGFs and inflammation of diseases. Application of specific modulatory molecules such as antibodies against pro-inflammatory FGFs/FGFRs like FGF23 will benefit for certain inflammation-related diseases.

## FGF signaling in metabolism

Among the 22 members of the FGF family, FGF15/19, FGF21, and FGF23 comprise the FGF19 subfamily that functions as endocrine hormones to regulate bile acid (BA), fatty acid, glucose, and mineral metabolism.

### FGF15/FGF19 in energy homeostasis

**Fig. 3:**
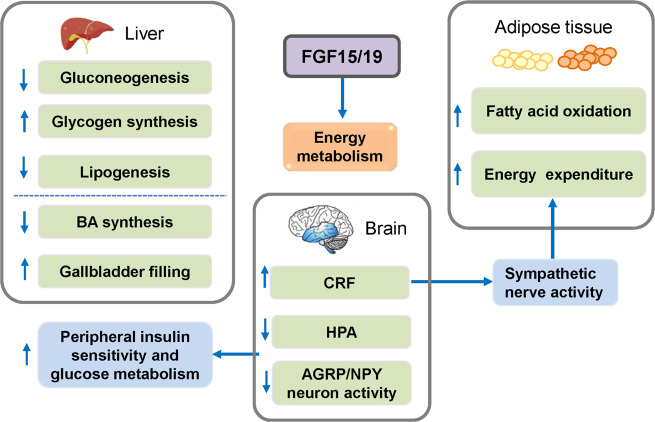
The regulation of FGF15/19 on energy metabolism. FGF15/19 regulates energy metabolism both peripherally and centrally. In the liver, FGF15/19 inhibits BA production and promotes gallbladder filling. As for lipid and glucose metabolism, FGF15/19 improves glycogen synthesis, but suppresses lipogenesis and gluconeogenesis. In the adipose tissue, FGF15/19 promotes energy expenditure and fatty acid oxidation. In the brain, FGF15/19 promotes the expression of CRF in the hypothalamus and stimulates sympathetic nerve activity, and then increases energy expenditure in the adipose tissue. Furthermore, FGF15/19 promotes peripheral insulin sensitivity and glucose metabolism by repressing HPA axis and AGRP/NPY neuron activity. AGRP agouti-related protein, BA bile acid, HPA hypothalamic-pituitary-adrenal, NPY neuropeptide Y

FGF15 and its human ortholog FGF19 (FGF15/19) are gut-derived circulating hormone that represses hepatic BA synthesis through FGFR4 and the coreceptor KLB complex.^528^ Furthermore, FGF15/19 also regulates global body energy and glucose homeostasis (Fig. 3).^529–531^

FGF15 is highly expressed in the ileum, jejunum, and duodenum of adult mice.^532,533^ FGF19 is expressed in human ileum and gallbladder epithelial cells,^534,535^ and is not detected normally in human liver.^536^ The expression and production of FGF15/19 are regulated by many factors, such as BAs, nutrition, and so on.^537,538^

#### Effect on liver and gallbladder

##### BA homeostasis

FGF15/19 negatively regulates BA synthesis. FGF19 treatment inhibits the expression of cholesterol 7α-hydroxylase (CYP7A1), the rate-limiting and major regulatory enzyme of BAs, by an autocrine/paracrine mechanism in hepatocytes.^529,539^ Deletion of *FGF15* in mice results in enhanced BA production by upregulating CYP7A1 expression in the liver, while FGF15 administration inhibits BA production by decreasing *CYP7A1* mRNA levels.^533^

The alternation of gallbladder filling and emptying regulates the bile flowing into the intestine. FGF15/FGF19 is required for gallbladder filling as evidenced by the absence of bile in the gallbladder of *FGF15* knockout mice, and FGF15 or FGF19 treatment leads to significant increase in gallbladder volume, which is partially caused by a cAMP-dependent relaxation of gallbladder smooth muscle.^540^

##### Hepatic glucose and lipid metabolism

Fed *FGF15* knockout mice showed decreased hepatic glycogen stores in the liver, and administration of FGF19 significantly promotes glycogen accumulation and protein synthesis in the liver of fasted mice, which is independent of insulin action.^541^ FGF15/19 also suppresses hepatic metabolic, such as the tricarboxylic acid cycle flux and gluconeogenesis, through inhibiting CREB-PGC-1α (cyclic AMP response element binding protein-peroxisome proliferator-activated receptor-γ coactivator-1α) signaling.^542^

FGF15/19 represses liver fat storage. FGF19 transgenic mice show decreased expression of lipogenic enzymes and liver triglyceride levels.^530^ FGF19 inhibits the expression of lipogenic enzymes and the insulin lipogenic action in rat primary hepatocytes through activating STAT3 signaling and repressing PGC-1β expression,^543^ and also enhances the expression of fatty acid oxidation-related proteins.^544^ Long-term treatment by FGF19 reduces liver lipid accumulation in vivo and protects liver from diet-induced steatosis.^545^

#### Effect on body energy and glucose homeostasis

FGF15/19 is beneficial for global energy balance. FGF19 transgenic mice have a significantly reduced fat mass resulted from increased metabolic rate that leads to enhanced energy expenditure, and do not become diabetic or obese when fed a high-fat diet (HFD).^530^ In HFD fed mice, FGF19 increases the metabolic rate simultaneously with an increased fatty acid oxidation, and alleviates the obesity in ob/ob mice.^529^ Adeno-associated virus (AAV) delivery of FGF15 and FGF19 reduces fat mass and increases energy expenditure in diet-induced obesity (DIO) mice, and FGF19 can also overt diabetes in db/db mice.^531^

In addition to the direct effects of FGF15/19 on body energy metabolism, FGF15/19 also regulates the energy and glucose metabolism by affecting brain after binding to FGFR4 and KLB in the brain.^358,546^ FGF19 activates ERK signaling in the hypothalamus.^547^ Intracerebroventricular (ICV) injection of FGF19 induces the sympathetic nerve activity to BAT and increases energy expenditure,^548^ and also improves peripheral insulin sensitivity and glucose metabolism by reducing hypothalamic agouti-related protein/neuropeptide Y neuron activity and activating of ERK1/2 signaling in obese and insulin-resistant states.^547^ Furthermore, FGF15/19 signaling in the central nervous system has an insulin-independent glucose-lowering effect. Acute ICV FGF19 injection reduces food intake and body weight, and improves glucose tolerance without changing plasma insulin levels.^546,549^ The suppressed hypothalamic-pituitary-adrenal (HPA) axis and subsequent decreased hepatic acetyl CoA level are responsible for mediating the insulin-independent, glucose-lowering effects of FGF19.^549^

### Metabolic role of FGF21

FGF21 is mainly expressed in the liver, adipose tissue and pancreas,^550,551^ and also expressed in the muscle.^552^ Under physiologic conditions, FGF21 in the blood is mostly derived from the liver.^551^ FGF21 activates FGF signaling by binding to FGFR1c and its coreceptor protein KLB in the liver, adipose tissue, and brain.^532^

**Fig. 4:**
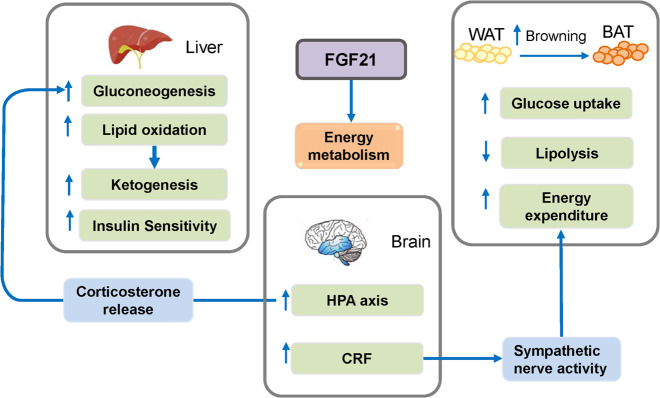
The regulation of FGF21 on energy metabolism. FGF21 regulates energy metabolism in peripheral and central manners. In the liver, FGF21 promotes gluconeogenesis and lipid oxidation, and thus improves ketogenesis and insulin sensitivity. In the adipose tissue, FGF21 simulates glucose uptake in both WAT and BAT and induces WAT browning, as well as promotes energy expenditure, while FGF21 inhibits lipolysis. In the brain, FGF21 stimulates the HPA axis, thus contributing to corticosterone release and ultimately promoting gluconeogenesis in the liver. Furthermore, FGF21 improves CRF expression in the hypothalamus and stimulates sympathetic nerve activity, and then promotes energy expenditure in BAT. BAT brown adipose tissue, CRF corticotropin-releasing factor, HPA hypothalamic-pituitary-adrenal, WAT white adipose tissue

FGF21 is a hormone regulating glucose and lipid homeostasis, and insulin sensitivity. FGF21 can cause weight loss, decrease plasma glucose and triglycerides level, and improve insulin sensitivity in obese and diabetic animal models without affecting total caloric intake.^553,554^ Mice with overexpressed *FGF21* resist to DIO. In both ob/ob and db/db mice,^553,554^ treatment of FGF21 decreased serum glucose and triglycerides to near normal levels. FGF21 regulates glucose and lipid metabolism mainly by affecting liver, adipose tissue, and brain (Fig. 4).

#### The effect on liver

Nutritional stresses, such as starvation, amino acid restriction, ketogenic, and HFD, can strongly induce the expression and release of FGF21 in liver.^555^

FGF21 decreases insulin resistance, enhances fat oxidation, and suppresses hepatic steatosis in the liver of DIO and ob/ob mice,^553,554^ which is related to the increased level of adiponectin in vivo.^556^ FGF21 participates in high-fat, low-carbohydrate ketogenic diet-induced triglyceride clearance, hepatic lipid oxidation, and ketogenesis. Downregulated hepatic FGF21 in ketogenic diet-fed mice altered the expressions of lipid and ketone metabolism-related genes in the liver, and leads to fatty liver, lipemia, and decreased serum ketone.^557^ FGF21 stimulates hepatic gluconeogenesis and ketogenesis in the liver during fasting and starvation^558,559^ by inducing the expression of PGC-1α.^559^ *FGF21* knockout mice fail to induce PGC-1α expression and have impaired gluconeogenesis and ketogenesis in response to a prolonged fast.^559^ However, the mechanisms for the regulation of FGF21 on liver metabolism need to be further explored.

#### The effect on adipose tissue

In addition to liver, adipose tissue is another source of systemic FGF21. White adipose tissue (WAT) stores energy, and brown adipose tissue (BAT) expends energy to generate heat through a process known as adaptive thermogenesis.^560^ FGF21 in WAT is induced by fasting/refeeding regimens and the thiazolidinedione drugs.^558^ FGF21 in BAT is induced by cold exposure.^561^

FGF21 stimulates glucose uptake in adipocytes in an insulin-independent manner through induction of *GLUT1* expression,^562^ and inhibits lipolysis of adipocytes.^563^ However, FGF21 stimulates lipolysis in WAT during starvation.^558^

The thermogenic activity of BAT and browning of WAT are important components of energy expenditure, which can be induced by FGF21.^554,564^ Cold exposure induces expression of mitochondrial uncoupling protein 1 (UCP1) in BAT. UCP1 uncouples oxidative phosphorylation, releasing chemical energy as heat.^565^ FGF21 improves the expression of UCP1 in WAT by upregulating PGC-1α protein level and promoting browning of WAT in adaptive thermogenesis.^559,566^ FGF21 knockout mice show diminished browning of WAT and a decreased adaption to chronic cold exposure.^566^ In addition, FGF21 also upregulates *UCP1* mRNA expression through CREB^567^ signaling, and induces phosphorylation of STAT3 to activate the oxidative metabolism in adipose tissues.^567^

FGF21 also promotes adipocyte differentiation and insulin sensitivity by stimulating peroxisome proliferator-activated receptor-γ (PPAR-γ) transcriptional activity through inhibiting its SUMOylation in WAT^568,569^ in DIO mice. FGF21 knockout mice show decreased WAT mass with reduced PPAR-γ activity, adipocyte size, and insulin sensitivity in DIO mice.^569^

#### The effect on brain

In addition to regulating liver and adipose tissue, FGF21 also involves in energy metabolism through regulating brain. FGF21 is not expressed in the central nervous system,^532^ but can cross the blood–brain barrier to enter into the brain.^570^ ICV injection of FGF21 in obese rats increases hepatic insulin sensitivity and energy expenditure.^571^ FGF21 improves the expression of neuropeptide corticotropin-releasing factor in the hypothalamus and stimulates sympathetic nerve activity, and then promotes energy expenditure in BAT.^572^ Furthermore, FGF21 activates the HPA axis for the release of corticosterone that stimulates hepatic gluconeogenesis.^359^

### The effect of FGF23 on mineral metabolism

**Fig. 5:**
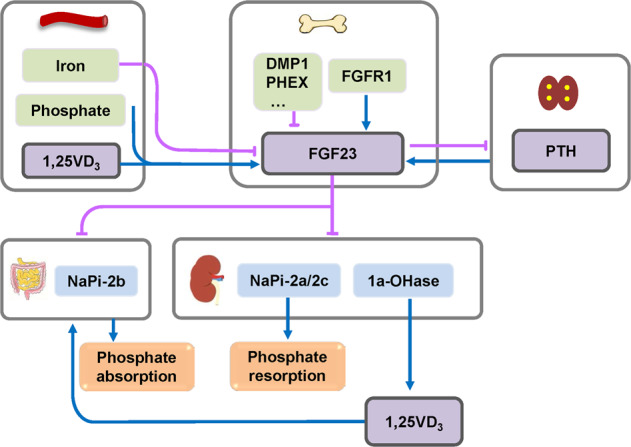
The regulation of FGF23 and its effect on phosphate homeostasis. The bone-derived FGF23 is regulated by several factors such as iron, phosphate, and 1,25(OH)_2_D_3_ and PTH in blood, as well as DMP1, PHEX, and FGFR1 in the bone. FGF23 downregulates serum phosphate. In the kidney, FGF23 reduces 1,25(OH)_2_D_3_ level and inhibits renal phosphate resorption by inhibiting the expression of NaPi-2a/2c. In the intestine, FGF23 inhibits phosphate absorption by reducing NaPi-2b expression or indirectly suppressing 1,25(OH)_2_D_3_. In the parathyroid, FGF23 inhibits PTH synthesis and secretion, and then contributes to its own negative feedback regulation. 1,25VD_3_ 1,25(OH)_2_D_3_, NaPi-2 type IIa sodium-phosphate co-transporter, PTH parathyroid hormone

FGF23 is mainly secreted by osteoblasts and osteocytes in bone tissue,^573^ and regulates systemic phosphate homeostasis and vitamin D metabolism through binding FGFR and the coreceptor α-Klotho complex in cell membranes of target tissues^574^ (Fig. 5).

#### The effect on metabolism of phosphate, sodium, and calcium

Clinical studies identified the important role of FGF23 in regulating phosphate metabolism. Mutations in an RXXR site in FGF23 lead to ADHR characterized by low serum phosphorus level, osteomalacia, and rickets, as well as short stature and bone pain.^128^ FGF23 is also the cause of tumor-induced osteomalacia and fibrous dysplasia because of its overexpression in tumors and osteogenic cells in fibrous dysplastic lesions.^575^ Furthermore, multiple *FGF23* gene mutations lead to reduced FGF23 level in patients, which is responsible for hyperphosphatemic familial tumoral calcinosis, a genetic disease characterized by hyperphosphatemia and tumor-like soft tissue calcifications.^576^ In mouse models, overexpression of *FGF23* in the liver, osteoblasts, or ubiquitously in mice lead to decreased serum phosphate concentration and rachitic bone.^177,577,578^

Phosphate homeostasis is simultaneously regulated by several organs, including the kidney, intestine, and bone.^579^ Type II sodium-dependent phosphate co-transporters (NPT2) are responsible for the absorption of extracellular phosphate.^580^ Type IIa sodium-phosphate co-transporter (NPT2a, NaPi-2a) is mainly expressed in the brush-border membrane of proximal tubules of the kidney.^581^ FGF23 inhibits renal phosphate reabsorption and leads to phosphate loss by inhibiting the expression of NaPi-2a/2c through binding to a FGFR1-α-Klotho coreceptor complex and activating ERK signaling.^582^ NaPi-2b is expressed in the luminal membrane of the ileum and regulates phosphate absorption in the intestine.^583^ FGF23 can reduce NaPi-2b level to inhibit phosphate absorption in the intestine.^584^ 1,25(OH)_2_D_3_ also promotes phosphate absorption in the intestine.^585^ FGF23 can reduce 1,25(OH)_2_D_3_ level by inhibiting 25-hydroxyvitamin D-1a-hydroxylase and increasing 25-hydroxyvitamin D-24-hydroxylase expression in the kidney,^586^ and then may indirectly suppress 1,25(OH)_2_D_3_-mediated intestinal phosphate absorption.

In addition, FGF23 also regulates the metabolism of sodium and calcium. FGF23 promotes sodium reabsorption by increasing the sodium chloride co-transporter expression in the distal renal tubules resulting in volume expansion and hypertension.^587^ FGF23 directly promotes calcium reabsorption in the kidney by regulating transient receptor potential vanilloid-5 channels in the distal renal tubules.^588^ Furthermore, 1,25(OH)_2_D_3_ promotes calcium absorption in the small intestine.^589^ PTH promotes calcium absorption in the kidney via increasing the 1,25(OH)_2_D_3_ level and accelerates calcium release from the bone by stimulating bone resorption. FGF23 can systematically regulate serum calcium by decreasing serum levels of 1,25(OH)_2_D_3_ and PTH.

#### The regulation of FGF23

FGF signaling participates in the regulation of FGF23. Several OGD patients caused by activating mutations of *FGFR1* present hypophosphatemia and increased serum level of FGF23.^132^ Inhibition of FGFR1 decreased *FGF23* mRNA expression in the bone.^590^ Integrative nuclear FGFR1 promotes *FGF23* transcription by activating the transcription factor CREB.^591^ HMW isoform of FGF2 (HMWFGF2), the ligand for nuclear FGFR1, stimulates *FGF23* expression.^592^ Transgenic mice with overexpression of *HMW FGF2* in immature and mature osteoblasts display increased FGF23 level, hypophosphatemia, and rickets.^592^

Some proteins regulating phosphate homeostasis are also expressed in osteoblasts and osteocytes, such as DMP1 and PHEX,^593,594^ and regulate FGF23 expression. Inactivating mutations in *DMP1* and *PHEX* lead to XLH (X-linked hypophosphatemic rickets) and ARHR (autosomal recessive hypophosphatemic rickets), respectively, accompanying with increased serum FGF23 level.^594,595^ Both *DMP1* and *PHEX* knockout mice exhibit hypophosphatemic rickets and increased *FGF23* expression.^594,596^ Although PHEX is a peptidase expressed in the bone, it can inhibit the expression FGF23 without regulating FGF23 degradation.^597^

Some circulating proteins also regulate FGF23 level. FGF23 is regulated by feedback loops, including the phosphate level, 1,25(OH)_2_D_3_, and PTH. Either dietary phosphate or administration of 1,25(OH)_2_D_3_ can increase the serum FGF23 level in humans and rodents,^598,599^ which depends on both translational and posttranslational regulation of FGF23.^600^ FGF23 inhibits PTH synthesis and secretion,^601,602^ and then contributes to its own negative feedback regulation. Patients with hyperparathyroidism have high FGF23 level,^603^ and some studies including cell culture experiments showed that PTH induces FGF23 expression in human and rodent cells through activating the orphan nuclear receptor Nurr1.^604,605^

Furthermore, iron can regulate FGF23 expression. Iron deficiency not only increases *FGF23* transcription,^606^ but also its cleavage.^607^ However, the detailed mechanism is still unclear.

## FGF signaling in tumors

A typical regulation of the FGF/FGFR system occurs in multiple human tumors, leading to the deregulated activation of ligand-dependent or -independent FGFR signaling.

### The expressions and mutations of FGF signaling molecules in tumors

FGF signal is highly related to the initiation and progression of several tumors including urothelial carcinoma, multiple myeloma, prostate cancer, and hepatocellular carcinoma (Table 2).

**Table 2:** Somatic GOF mutations of FGFRs in cancers

Gene	Type	Site	Cancers
*FGFR1*	Amplification		Breast cancer (ER+)
			Gastric cancer
			Lung cancer (SCC, SC)
			Ovarian cancer
			Urothelial cancer
	Fusion	FGFR1-TACC1	Glioblastoma
		BCR-FGFR1, CNTRL-FGFR1, ZMYM2-FGFR1, etc.	MPN
	Mutation	N546K	Ewing sarcoma
		N546K, K656E	Glioblastoma
*FGFR2*	Amplification		Breast cancer (TNBC)
			Gastric cancer
	Fusion	FGFR2-AFF3, FGFR2-CASP7	Breast cancer
		FGFR2-BICC1, FGFR2-PPHLN1, etc.	Cholangiocarcinoma
		FGFR2-CIT	Lung cancer
	Mutation	R203C, N549K, K659N	Breast cancer
		S252W, P253R, N549K, K659E	Endometrial cancer
		S252W, P253R, K659E	Lung cancer
*FGFR3*	Amplification		Ovarian and urothelial cancers
	Fusion	FGFR3-TACC3	Glioblastoma and lung cancer
		ETV6-FGFR3	Lymphoma
		t(4;14) (p16;q32)	Multiple myeloma
		FGFR3-BAIAP2L1, FGFR3-JAKMIP1, FGFR3-TACC3	Urothelial cancer
	Mutation	R248C, S249C, G370C, Y373C, G380R, K650M	Gallbladder cancer
		R248C, S249C, G370C, K650E	Lung cancer
		R248C, Y373C, K650E/M	Multiple myeloma
		R248C, S249C, G370C, S371C, Y373C, N540S, K650E/M	Urothelial cancer
*FGFR4*	Mutation	N535K, V550E	Rhabdomyosarcoma

#### Expressions of FGFs

*FGF5* is overexpressed in breast cancer tissue.^608^ Guo et al.^609^ reported that *FGF6* was significantly decreased in non-metastatic liver cancer lesion tissues and increased in metastatic liver carcinoma tissue. FGF7 is expressed in normal mucosal gland epithelium and in stromal fibroblasts, and FGF7 protein levels were elevated in gastric inflammation and gastric adenocarcinoma.^610^ Overexpression of FGF8 in prostate cancer is highly related to the decreased patient survival and persists in androgen-independent disease.^611^ FGF8, as cell growth regulator, can mediate the tumor suppression effect of Annexin-A7 in prostate tumorigenesis.^612^ *FGF9* is expressed in many non-small cell lung carcinoma (NSCLC) primary tumors and derived cell lines. The NSCLC patients with high *FGF9* expression had shorter overall survival.^613^ Aberrant signaling of FGF10 through FGFR2b, and in some instances FGFR1b, contributes to the progression of a number of human cancers, including breast cancer, prostate cancer, and pancreatic adenocarcinoma, as well as gastric cancer (GC), skin cancers, and lung squamous cell carcinomas.^614^ *FGF12* gene was overexpressed in esophageal squamous cells.^615^ *FGF13* was highly upregulated in aggressively metastatic breast tumors and pancreatic endocrine tumors.^616^ *FGF14* was preferentially methylated in colorectal cancer.^616^ The expression of *FGF16* is markedly increased in ovarian tumors.^617^ FGF17 is overexpressed as a potential mediator of FGF8 function in human prostate cancer.^618^ In genomically stable and chromosomal instable subtypes of GC, FGF18 was overexpressed with relevance to poor survival.^619^ FGF18/FGFR3IIIc was upregulated and could drive growth of tumor cell in CD44^+^ subpopulation of colon adenoma cells.^620^ Aberrant signaling through FGF19 and its receptor FGFR4 seems to be the oncogenic driver for a subset of human hepatocellular carcinoma (HCCs) and is associated with poor prognosis.^621^ Ectopic expression of *FGF20* in NIH 3T3 cells rendered the cells transformed in vitro and tumorigenic in nude mice.^622^ The mRNA level of *FGF20* was upregulated in adenomas in mice and FGF20 is found to be a critical element in Wnt signaling-induced oncogenesis.^623^ Huang et al.^624^ found that overexpression of *FGF21* delayed the appearance of diethylnitrosamine-induced liver tumors and proposed that FGF21 might delay development of adenomas through activation of resident hepatocyte FGFR4 at early time. Liu et al.^625^ demonstrated that *FGF22* expression was tightly associated with the poor overall survival. FGF23 is present at an increased level and promotes the progression of prostate cancer.^626^

#### Mutations of FGFs and FGFRs in tumors

The risk of relapse in the subgroup of progesterone-receptor-negative patients of breast tumors was five times greater for those with *int-2/FGF3* amplification than for those without this alteration.^627^ High-throughput tissue microarray analysis showed that gene amplifications of *FGF3* and *FGF4* were observed in urinary bladder cancer.^628^ Kim and his colleagues^629^ revealed that three SNPs in the *FGF23* gene (rs11063118, rs13312789, and rs7955866) were associated with an increased risk of prostate cancer. Mutations of *FGFRs* are commonly observed in many tumors, including the breast cancer, lung cancer, liver cancer, GC, uterine cancer, and bladder cancer.^630^ *FGFR1* amplification is one of the most common focal amplifications in breast cancer.^631^ *FGFR1* amplification was observed in 32% of small cell lung cancer samples.^632^ A single somatic *FGFR1* mutation (c.C754A p.P252T) was also detected in a bronchoalveolar cancer.^633^ Constitutional and somatic FGFR1 alterations were frequently observed in dysembryoplastic neuroepithelial tumor (DNET) and played a key role in the pathogenesis of DNET.^634^ FGFR2 amplifications have been observed in nearly 10% of GCs, playing a critical role in the proliferation and survival of GC cell.^635^ GC cell lines with *FGFR2* amplifications were highly sensitive to FGFR inhibitors.^636^ Dutt et al.^637^ reported that somatic mutations of *FGFR2* were present in 12% of endometrial carcinomas, and inhibition of FGFR2 kinase activity in endometrial carcinoma cell line bearing such FGFR2 mutations could inhibit its transformation and survival, implicating FGFR2 as a novel therapeutic target in endometrial carcinoma. *FGFR2* fusions were reported to be present in up to 13% of liver cancers such as intrahepatic cholangiocarcinoma.^638,639^ *FGFR2* amplifications occur in triple-negative breast cancer, and are associated with high sensitivity to FGFR inhibitors.^640^ FGFR2 is shown to be associated with a higher risk of sporadic post-menopausal breast cancer.^641^ Amplifications of *FGFR3* have been described rarely in cancer, while activation of FGFR3 by mutation was quite common.^642^ FGFR3 alterations (mutations or translocation) are among the most frequent genetic events in bladder carcinoma. Single-nucleotide substitution mutations of FGFR3 were present in 35% of bladder carcinomas.^643^ The mutations of FGFR3 could lead to an aberrant activation of FGFR3 signaling, conferring an oncogenic dependence, while inhibition of FGFR3 signaling decreased cell viability in vitro and tumor growth in vivo.^644^ *FGFR3* mutations were also identified in cervical cancers,^645^ multiple myeloma,^38^ prostate cancer,^646^ testicular tumors,^647^ and lung adenocarcinoma.^648^ *FGFR1-*3 gene fusions have been observed in breast cancer to occur with multiple gene partners (i.e., *TACC1-3, BAIAP2L1, AFF3, SLC45A3, and AHCYL1*).^640^ A very low level of amplifications of *FGFR3* and *FGFR4* were detected in breast cancer.^649^ Mutations in *FGFR4* in human rhabdomyosarcoma (RMS) could lead to its activation and contribute to RMS progression as an oncogene.^650^ The mutation of *FGFR4* gene transcript in MDA-MB-453 mammary carcinoma cells lead to the substitution of glycine by arginine at position 388, which increased cell motility. The FGFR4 Arg388 allele was related to the metastasis of colon cancer in patients.^651^

### FGFs and FGFRs in tumorigenesis

FGF/FGFR signaling is involved in the major steps of tumor progression, including cancer cell survival and proliferation, angiogenesis, invasion, and metastatic dissemination and response to therapy.

#### FGFs and FGFRs in tumor growth

The expression of FGF4 was increased in germ cell tumors, especially in non-seminomas, which could promote malignant growth of cultured embryonal carcinomas by targeting all-*trans*-retinoic acid.^652^ FGF2 can induce breast cancer growth through ligand-independent activation and stimulate the MYC gene expression through recruitment of ERα and PRB$\delta$4 isoform to MYC regulatory sequences.^653^ The results from Betsuyaku, T.’s group showed that the FGF2 aptamer that can block FGF2 activity could inhibit the growth of FGF2-FGFR pathway-dependent lung cancer cells.^654^ Increased expression of *FGF4* in ovarian cancer stem-like cells/cancer-initiating cells is involved in the upregulating tumor initiation capacity of fibroblasts.^655^ Fang et al.^656^ demonstrated that miR-188-5p suppressed the tumor cell proliferation and metastasis by directly targeting *FGF5* in HCC. The neutralizing antibody to FGF8b could significantly inhibit cell growth of prostate cancer.^611^ In mouse Leydig tumor cells, FGF9/FGFR2 signaling can increase its proliferation by activating ERK1/2, Rb/E2F1, and cell-cycle pathways.^657^ Downregulation of *FGF18* suppressed the tumor formation abilities, induced G1-phase cell-cycle arrest and enhanced anticancer drug sensitivity.^619^ The antibody of FGF19 could inhibit the growth of colon tumor xenografts in vivo and effectively prevent HCCs in *FGF19* transgenic mice, suggesting that the inactivation of FGF19 could be beneficial treatment for cancers and other malignancies involving interaction of FGF19 and FGFR4.^658^ Low concentration exogenous FGF19 promoted the growth of prostate cancer cells, while inhibition of FGF19 in prostate cancer cells could decrease proliferation in vitro and tumor growth in vivo.^629^ FGF9 greatly contributes to Pregnane X receptor-mediated tumor aggressiveness in humans and mice.^659^ In endoplasmic reticulum stress-induced HCC cells, *FGF19* overexpression promoted cell survival and increased resistance to apoptosis, whereas *FGF19* silencing counteracted these effects.^660^ *FGF19* gene amplification has been found to be corresponding with an increased dependency upon FGF19/FGFR4 autocrine signaling mediated by ERK/AKT-p70S6K-S6 activation in head and neck squamous cell carcinomas.^661^ FGF23 enhances the proliferation, invasion, and anchorage-independent growth of prostate cancer cell lines in vitro, while *FGF23* KD also decreases tumor growth in vivo.^626^ Activation of FGFR1 leads to rapid tumor growth as a result of increased proliferation in prostate cancer cells.^662^ FGFR2 promotes breast cancer tumorigenicity by maintaining tumor-initiating cells.^663^ FGFR3 is overexpressed in the early stages of bladder cancer, and targeting the extracellular domain of FGFR3 with human single-chain Fv antibodies could suppress the proliferation of bladder carcinoma cell line.^664^

#### FGFs and FGFRs in the invasion and migration tumors

Henriksson et al.^665^ reported that colorectal cancer cells activate adjacent fibroblasts, which results in enhanced FGF1/FGFR3 signaling and subsequent increased invasion of tumor cells. Abrogation of the nuclear translocation of *FGFR1* and *FGF2* in pancreatic cancer cells significantly inhibit cancer cell invasion.^88^ FGF7/KGF could trigger cell transformation and invasion of immortalized human prostatic epithelial PNT1A cells.^666^ FGF7/FGFR2/THBS1 promotes the invasion and migration in human GC.^667^ FGF9 secreted by cancer-associated fibroblasts is considered as a possible mediator by promoting the anti-apoptosis and invasive capability of GC cells.^668^ FGF10/FGFR2 signal can significantly promote the cell migration and invasion in pancreatic cancer.^669^ FGF16 enhanced the invasion of SKOV-3 ovarian cancer cells through activation of MAPK signaling pathway.^617^ The members of FGF8 subfamily including FGF8, FGF17, and FGF18 are involved in autocrine and paracrine signaling in HCC and enhance the survival of tumor cells, tube formation, and neoangiogenesis.^670^ FGF18 has been reported to control the migration, invasion, and tumorigenicity of ovarian cancer cells through NF-κB activation, which increased the production of oncogenic cytokines and chemokines.^671^ FGF9 greatly contributes to Pregnane X receptor-mediated tumor aggressiveness in humans and mice.^659^

#### FGFs and FGFRs in tumor angiogenesis

The onset of angiogenesis is a discrete step that occurs at any stage of tumor progression. FGF ligands and receptors promote angiogenesis in a variety of tumors.^672^ Wang and Becker^673^ showed that delivery of an episomal vector containing antisense *FGF2* or *FGFR1* cDNA could completely prevent the growth of tumors partially through the blockage of angiogenesis in the human melanoma grown as a subcutaneous tumor model in nude mice. FGF2 can induce tumor growth and neovascularization in vivo.^674^ FGF2 and MMP2 may cause increased angiogenesis and invasion of bone marrow plasma cells in several unidentified monoclonal gamma globulin disease and multiple myeloma cases.^675^ FGF binding protein can be used as an angiogenesis conversion molecule in human tumors via promoting the release of biologically active FGF2 and leading to tumor growth.^676^ The type 1 repeats of thrombospondin-1 (TSP1) can block angiogenesis driven by FGF2 or vascular VEGF and inhibit tumor growth.^677^ IL-10 blocks the proliferation of microvascular ECs induced by VEGF and FGF2 in vitro and has a direct effect on preventing angiogenesis in human lymphomas.^678^ It has been reported that the average serum FGF2 level was significantly increased (~7 times) in testicular cancer patients, and the expression level of FGF2 was also significantly increased in tumor biopsies.^679^ Targeting the mRNA of early growth response (*EGR1*) an upstream of FGF2, can inhibit the expression of EGR1 protein and block tumor angiogenesis.^680^ Human melanoma cell survival and growth depend on autocrine action of FGF2.^681^ In addition, neutralized FGF2 with antibodies could block the angiogenesis in melanoma cell lines transplanted nude mice models.^682^ In addition, FGF2 is shown to be involved in angiogenesis in the formation of pituitary tumors.^683^ FGF1 can cause increased angiogenesis that contributes to the poor survival rate of patients with advanced serous ovarian cancer.^684^ Two angiogenic factors PDGF-BB and FGF2 in tumors can synergistically promote the neovascularization and metastasis in murine tumor model.^685^ Targeted inhibition of PDGF receptors can downregulate the expression of FGF2 and epithelial growth factor FGF7, thereby reducing angiogenesis.^686^

## Therapeutics and strategies for targeting FGF signaling

FGF signaling plays critical roles in tissue/organ development and homeostasis, and dysregulated FGF signaling has been found in a variety of diseases and injuries (see above). It is a promising therapeutic strategy for these diseases/injuries by modifying or correcting the aberrant FGF signaling. So far, FGF-based therapeutics are largely classified into three classes, including enhancing FGF signaling therapeutics, blocking FGF signaling therapeutics, and gene therapy.

### Enhancing FGF signaling therapeutics

FGFs are involved in numerous pathophysiological processes;^1^ recombinant FGF or FGF analogs have been developed as first-generation strategies to augment the beneficial effects of FGFs/FGFRs (shown in Table 3).

**Table 3:** Therapeutics targeting FGF signaling

Class	Drug	Targets	Diseases	Drug development
Recombinant FGFs or FGF analogs	rmFGF1	FGF1 receptor	T2DM	Preclinical
	rhFGF1	FGF1 receptor	T2DM	Preclinical
	rhFGF2 (trafermin)	FGF2 receptor	Skin ulcers Periodontitis	Approved Japan P3 (NCT01015404)
	FGF7 (palifermin)	FGF7 receptor	Oral mucositis	Approved USA
	FGF10 (repifermin)	FGF10 receptor	Mucositis	Clinical trials was terminated in 2004
	rhFGF18 (sprifermin)	FGF18 receptor	Osteoarthritis	P2 (NCT01919164)
	FGF19-4/5/6	FGF19 receptor	Tumorigenicity	Preclinical
	FGF19 variant (FGF19v)	FGF19 receptor	Mitogenic	Preclinical
	NGM282 (M70)	FGF19 receptor	T2DM PSC	P2 (NCT01943045) P2 (NCT02704364)
	Obeticholic acid and Px-104	FGF19 receptor	Primary/secondary bile acid malabsorption Obesity NAFLD	P2 (NCT01585025) P2 (NCT01625026) P2 (NCT01265498) P2 (NCT01999101)
	LY2405319	FGF21 receptor	T2DM	P1 (NCT01869959)
	FGF21variant (PEG-FGF21^G71C^, Fc-FGF21(RG))	FGF21 receptor	T2DM	Preclinical
	PF-05231023 (CVX-343)	FGF21 receptor	T2DM	P1 (NCT01285518)
Non-selective TKIs	Lucitanib (E3810)	FGFR1/2, VEGFR1/2/3, and PDGFRα/β	Cancer with FGFR alteration	P2 (NCT02747797) P3 (NCT00165672)
	Nintedanib (BIBF1120)	FGFR1/2/3, VEGFR1/2/3, and PDGFRα/β	Cancer with FGFR alteration	Submitted P3
	Dovitinib (CHIR258 or TKI258)	VEGFR1/2/3, FGFR1/2/3, PDGFRβ, c-Kit, RET, TrkA, CSF-1R, and FLT3	Cancer with FGFR alteration	P2 (NCT01719549) P2 (NCT01732107)
	Regorafenib			P2 (NCT01929616)
	Brivanib			P2 (NCT03516071)
	Ponatinib			Approved for market
	Lenvatinib	FGFR1/2/3	Cancer with FGFR	P2 (NCT03609359)
	Pazopanib		alteration	P2 (NCT01253369)
	Orantinib			P3 (NCT01465464)
	Sunitinib			P2 (NCT00768144)
	Cediranib			P3 (NCT00399035)
Selective TKIs	AZD4547	FGFR1/2/3	Cancer with FGFR alteration	P2 (NCT01824901) P2 (NCT01791985) P2 (NCT02824133) P2 (NCT01213160)
	BGJ398 (NVP-BGJ398)	FGFR1/2/3	Cancer with FGFR alteration	P2 (NCT01975701) P2 (NCT02150967) P2 (NCT02160041)
	JNJ-42756493 (erdafitinib)	FGFR1/2/3/4	Cancer with FGFR alteration	P2 (NCT02365597) P2 (NCT02699606)
	LY287445, Debio-1347, TAS-120, and BAY-1163877	FGFR1/2/3/4	Cancer with FGFR alteration	Preclinical
Neutralizing monoclonal antibodies (mAbs)	KRN23	FGF23	XLH	P3 (NCT02537431)
	Bemarituzumab (FPA144)	FGFR2b	Neoplasms	P1 (NCT02318329)
	BAY1179470	FGFR2	Neoplasms	P1 (NCT01881217)
	MFGR1877S	FGFR3	Neoplasms	P1 (NCT01122875)
	hIgG1-1A2	FGF2		
	GAL-F2	FGF2		
	3F12E7	FGF2		
	KM1334	FGF8b	Neoplasms	Preclinical
	FGF10 mAb	FGF10		
	FN1 and FC1	FGF23		
	R1MAb1	FGFR1		
FGF traps	FP-1039 (GSK3052230)	FGF1/2/4	Neoplasms	P1 (NCT01868022)
	SM27	FGF2	Angiogenesis	Preclinical
	NSC12	FGF2	Lung tumors	Preclinical
	sFGFR2IIIc (S252W)	FGFR2	AS	Preclinical
	sFGFR3	FGF2/9/18	Chondrodysplasia	Preclinical
	Peptide P3	FGFR3	Chondrodysplasia	Preclinical
Gene therapy	XRP0038 (NV1FGF)	FGF1 receptor	Peripheral vascular diseases	P2 (NCT00566657)
	Expression of *FGF18* cDNA	FGF18 receptor	Murine models	Preclinical
	AAV9-*Fgfr2*-shRNA	*Fgfr2-P253R* allele	AS	Preclinical
	CRISPR/Cas9	*Fgfr3*-G374R	Achondroplasia	Preclinical

*T2DM* type 2 diabetes mellitus, *PSC* primary sclerosing cholangitis, *NAFLD* non-alcoholic fatty liver disease, *XLH* X-linked hypophosphatemia, *AS* Apert syndrome, *P1* phase I clinical trial, *P2* phase II clinical trial, *P3* phase III clinical trial

Canonical FGFs, encoded by *FGF1*, *FGF4*, *FGF7*, *FGF8*, and *FGF9* subfamily gene, by binding to heparan sulfate proteoglycans largely exert their effects locally.^1^ A single injection of mouse recombinant FGF1 causes potent, dose- and insulin-dependent glucose lowering in diabetic mice without hypoglycemia.^687^ Recombinant human FGF1 (rhFGF1) is also able to normalize blood glucose in diabetic mice.^687^ In addition, trafermin (rhFGF2) has been supported for their use in the patients with skin ulcers,^688^ and in phase III clinical trial, trafermin was further approved for its application in patients with periodontal surgery. Palifermin, a truncated form of FGF7, has been approved for the treatment of patients with oral mucositis.^688^ In pediatric patients, palifermin may provide advantage to prevent chemotherapy-induced mucositis.^689^ Repifermin, a truncated form of FGF10, with the pharmacological effects similar to that of FGF7, promotes the healing of ulcerated oral and intestinal mucosal tissue, and reduces the complications in preclinical tests.^690^ However, the clinical trials about the effect of repifermin on mucositis were terminated in 2004 as no effective evidence for reducing the incidence or severity.^691^ In addition, rhFGF18 have been approved for treating OA and cartilage injury of the knee in phase II clinical trial.^266^

Endocrine FGFs, encoded by *FGF19* subfamily gene, which bind and activate FGFRs with the Klotho family protein, regulate a wide range of metabolic processes.^692^ Based on the structure–function principle, separating mitogenic and metabolic activities of FGF19 through mutagenesis of five N-terminal and heparin-binding regions of FGF19 yielded a series of FGF19 variants, which retain the beneficial metabolic effects, while reduce the side effects of FGF19 on tumorigenicity.^693^ In addition, a new constructed FGF19 variant (25-194 of FGF19 and 1-20 of FGF21), impaired in activating FGFR4 and still had beneficial effects on glucose and lipid metabolism.^694^ These studies provide a strategy for engineering FGF19 as a potential therapy for related diseases/injuries. These variants were found to be devoid of BA regulatory activity. However, another FGF19 variant NGM282 (M70) retains the beneficial BA metabolism effects, while is devoid of murine mitogenic activity by inactivating the STAT3 pathway.^695^ To date, M70 as one FGF19 variant was studied through phase II clinical trials for their use in patients with primary sclerosing cholangitis and diabetes mellitus. In addition, several FGF19-inducing strategies (farnesoid X receptor agonists) such as obeticholic acid and Px-104 were tested through phase II clinical trials and provided with further support for their use in the patients with primary/secondary BA malabsorption and nonalcoholic fatty liver disease, respectively.

Several strategies have been used to optimize the “druggability” of FGF21. LY2405319, a novel FGF21 variant, was reconstructed by introducing an additional disulfide bond firstly in the C terminal of FGF21 by mutations (L118C, A134C), and then further optimized by deleting His-Pro-Ile-Pro in the N terminal of FGF21 along with a mutation to replace the major site of O-linked glycosylation (Ser167Ala). Subcutaneous administration of LY2405319 in DIO mice exhibited a potency similar to FGF21, resulting in decreased plasma glucose along with a reduction in body weight.^696^ To date, LY2405319 has been tested through phase I clinical trial to reduce body weight and fasting insulin, and is noteworthy in improving dyslipidemia in patients with type 2 diabetes mellitus.^697^ Another FGF21 variant is reconstructed through the introduction of *p*-acetyl phenylalanine into the N-terminal residue of rhFGF21 for the attachment of PEG (PEGylated rhFGF21).^698^ PEGylated rhFGF21 has the ability to normalize insulin-mediated glucose utilization in diabetic murine models,^699^ but exhibits remarkably lower bioactivity than FGF21, along with induction of renal vacuole formation.^700^ Song et al.^701^ further optimized FGF21 by introducing G71C mutation to generate the mimetic PEG-FGF21^G71C^, which exhibits increased half-life. Subsequently, an alternative strategy was adopted to yield Fc-FGF21 by fusing Fc fragment of human IgG1 to the N-terminal end of FGF21 to improve the pharmacokinetic properties of FGF21, which exhibited a prominently increased half-life compared to the native FGF21.^702^ Since, the C-terminal region of Fc-FGF21, especially between Pro171 and Ser172, was rapidly degraded, Pro171Gly mutation was introduced to retain biological activity, while eliminate the proteolytic degradation.^703^ Moreover, FGF21 has the additional concern of forming aggregates during protein production. By combining Pro171Gly and Leu98Arg mutations into one molecule, a novel variant named Fc-FGF21 (RG) was generated with resistance to aggregation and proteolysis.^703^ Another approach to improve plasma half-life is to fuse FGF21 to a scaffold monoclonal antibody (mAb).^704,705^

### Blocking FGFs signaling therapeutics

Given that a variety of human diseases and injuries caused by excessive FGF signaling. So far, the measures blocking FGF signaling can be generally classified to TKIs, neutralizing mAbs, and FGF traps.

#### TKIs

##### Nonselective TKIs

Nonselective TKIs have been developed as first-generation strategies to blocking FGFs signaling. These TKIs have the benefit of concurrently targeting tumor proliferation and angiogenesis, while also displaying a remarkable effect against FGFR signaling pathways, together with a multiplicity of adverse effects that limit their use in clinic.

Lucitanib (E3810) is a triple TKI, which targeting FGFRs, VEGFRs, and PDGFRs. E3810 showed a promising efficacy and a manageable side effect in patients with both FGF-aberrant or angiogenesis-sensitive tumor types.^706^ Until 2018, E3810 were completed phase II clinical trials, which inhibits the growth of tumor by antiangiogenesis.

Nintedanib (BIBF1120) is another novel triple angiokinase inhibitor, with less activity against SRC, RET, and FLT3.^707^ BIBF1120 competitively binds to the ATP-binding pocket of these receptors, and blocks the intracellular signaling critical for the proliferation and survival of angiogenesis-related cells.^707^ Up-to-date, BIBF1120 has been approved for the treatment of pulmonary fibrosis and as a second-line therapy for NSCLC in combination with docetaxel. Phase III clinical trials are still ongoing to study the response of patients selected for specific FGFR alterations.^708^

Dovitinib (CHIR258 or TKI258) is an oral ATP-competitive multikinase inhibitor that targets FGFRs, VEGFRs, and PDGFRβ.^709^ TKI258 has a promising inhibitory activity in cell lines with FGFR translocations or amplification.^710^ In phase II trials, TKI258 can stabilize disease in multiple myeloma bearing t (4;14) translocation by blocking FGFR3 activity.^711^

Beyond that, several other nonselective TKIs are shown in Table 3, which have been developed and are in preclinical and clinical evaluation.^712^ However, these nonselective TKIs induce a series of side effects: cardiotoxicity or proteinuria on account of the concurrent VEGFR inhibition, as well as cutaneous reactions, digestive disorders, and gastrointestinal disease, for example.^153^

##### Selective TKIs

To overcome the off-target effects, second-generation selective FGFR TKIs have been developed.

AZD4547 is a potent reversible TKI specific for FGFRs.^713^ Of note, AZD4547 is able to sharply diminish cancer stem-like cells by inducing MET via MEK/ERK pathway downstream of FGFR signaling.^714^ In addition, administered AZD4547 prominently impaired ductal branching and stem cell-like characteristics in mammary epithelial cell and spontaneous tumor cells.^715^ In phase I/II trials, AZD4547 further showed promising inhibitory activity in models of cancer with FGFR alteration.

BGJ398 (NVP-BGJ398) is a selective reversible ATP-competitive inhibitor targeting FGFRs, which showed superior potency to ponatinib and dovitinib, and exerted a more potent therapeutic effect against chemotherapy-refractory cholangiocarcinoma containing FGFR2 fusions.^638^ Of note, in phase I/II trials, BGJ398 promoted tumor reduction in patients with FGFR-related advanced solid tumors.^716^

JNJ42756493 (erdafitinib) with potent TKI activity can target all FGFRs, which suppresses phospho-FGFR and phospho-ERK resulting in dose-dependent antitumor activity.^717^ Further in phase I/II trials, the administered erdafitinib has an inhibitory activity in patients with advanced solid tumors characterized by FGFR translocations or FGFR3–TACC3 fusions.^717–719^

Other selective TKIs are shown in Table 3, and showed promising results in preclinical and clinical evaluation on different oncotypes.^712^

Unfortunately, drug resistance limits the success of TKIs with mutations at the “gatekeeper” residue, leading to tumor progression. Structural analyses showed that the FGFR1 “gatekeeper” mutation (V561M) can induce a potently increased autophosphorylation, in part, by a network of interacting residues forming a hydrophobic spine to stabilize the active conformation. Further kinetic assays established that V561M confers significant resistance to E3810, while it retains affinity for AZD4547 due to a flexible linker that allows multiple inhibitor binding modes.^720^ In addition, JNJ42756493 binds to the ATP pocket of the FGFR1 KD with unique structural conformations, and its inhibitory efficacy is reduced by 200-fold in the FGFR3 “gatekeeper” mutation (V555M), while an increase in efficacy for TKI258.^721^ In contrast, some FGFR2 “gatekeeper” mutations drive acquired resistance to TKI258 by causing steric hindrance to the binding of the TKI to the receptor (such as N550K, E566G, and K660E) or by stabilizing the active conformation of the kinase (V651I).^722^ Moreover, multiple recurrent patients have point mutations in the FGFR2 KD at progression, and each mutation drives acquired resistance to BGJ398, and was surmountable by structurally distinct FGFR inhibitors.^723^ Thus, designing inhibitor with flexibility to overcome drug resistance may be an vital way for exploiting effective inhibitor against mutation.

#### Neutralizing mAbs

When compared to TKIs, neutralizing mAbs have unique advantages of low toxicity due to the absence of off-target effects.

Burosumab (formerly KRN23) is a fully human IgG1 mAb that binds to and blocks the biologic activity of FGF23. Injection of Burosumab normalized both phosphate and vitamin D concentrations in hypophosphatemia mouse models.^724^ In 2019, phase II clinical trials for Burosumab was completed and provided support for its use for XLH.

Bemarituzumab (FPA144) is a rhIgG1 mAb that specially binds to the IgG III region of the FGFR2b receptor isoform to prevent ligand binding and downstream signaling activation. In phase I clinical trial, a single dose of FPA144 was conducted in gastroesophageal adenocarcinoma (GEA) patients with FGFR2b overexpression, which remarkably inhibited GEA growth.

MGFR1877S is an mAb targeting FGFR3 by hampering its dimerization, which is well tolerated with low toxicities in patients with multiple myeloma and solid tumors in phase I clinical trials.^708^

Beyond that, there are other mAbs awaiting further confirmation in preclinical and clinical testing, such as BAY1179470, hIgG1-1A2, GAL-F2, 3F12E7, KM1334, FGF10 mAb, FN1, FC1, and R1MAb1, as detailed in Table 3.

#### FGF traps

An alternative strategy to modulate the activity of the FGF/FGFR signaling is to use the molecules able to bind and neutralize multiple FGF ligands. This strategy represents a novel path for the development of FGF traps.

FP-1039 (GSK3052230) is an FGF ligand trap that binds and neutralizes multiple FGFs and thus inhibits the activation of FGFR1. In preclinical trials, FP-1039 blocked FGF2-stimulated tumor cell proliferation and inhibited tumor growth in xenograft models.^672^ In phase I clinical trials, associated with paclitaxel and carboplatin, or docetaxel, intraperitoneal injection of FP-1039 was well tolerated in patients with solid malignancies.^725^ However, FP-1039 does not effectively inhibit endocrine FGFs (FGF19, FGF21, and FGF23).^726^ Therefore, FP-1039 has the potential to effectively block the neoplasms or advanced cancer-promoting FGFs, with less toxicity compared to small molecules such as FGFR kinase inhibitor.

The development of FGF trap agents has also relied on the structural characterization of the interactions of FGFs with their natural “interactome,” including thrombospondin-1 (TSP1), HSPGs, and pentraxin-3 (PTX3).^727^ Structural analysis of the complex between FGF2 and TSP1 identified a new small-molecule SM27 that inhibits FGF2-induced angiogenesis through binding to FGF2.^728^ Similar to the integrative TSP1, SM27 perturbs FGF2 dynamics in distant regions, including the FGFR1 binding site, by binding the heparin affinity site of FGF2, thus preventing FGF2 binding to HSPG and FGFR1.^729^ Therefore, SM27 acts as a dual direct and allosteric inhibitor of the binding between FGF2 and its receptors, which has unique benefits for the development of novel cancer drug. In addition, structural analysis of the complex between FGF2 and the N terminal of PTX3^730^ identified an acetylated pentapeptide ARPCA as the minimal FGF2 binding peptide that inhibits FGF8b-induced angiogenesis.^731^ Besides, based on pharmacophore modeling of the ARPCA/FGF2 interaction, NSC12 was identified as multi-FGF trap that can participate in the formation of the HSPG/FGF2/FGFR1 ternary complex. In tumor models, administration of NSC12 can block the growth, angiogenesis, and metastasis of FGF-dependent lung tumors.^732^

In addition, a soluble FGFR2 mutant with S252W (sFGFR2IIIc (S252W)) was found to partially alleviate the AS in mice by alleviating the premature closure of coronal suture in cultured calvarias and transgenic mice.^733,734^ Moreover, sFGFR3, a recombinant protein, acts as a FGFR trap to prevent FGF ligand binding to FGFR3. In ACH mice, subcutaneous injection of sFGFR3, to compete with endogenous FGFR3 ligands, showed a dose-dependent rescue of chondrodysplasia phenotypes.^735^ Besides, in TD II model, administration of peptide P3 with the ability to downregulate the activity of FGFR3 rescues the lethal phenotype and partially restores the structural distortion of growth plates.^736^

### Gene therapy

At present, gene therapy is inevitable, especially in the era of precision medicine. Expression of *FGF18* by AAV-mediated gene transfer in the pinnae of nude mice resulted in a noteworthy increased thickness due to an *FGF18*-mediated increase in chondrocyte proliferation and ECM production.^238^ Conditional expression of *FGF18* in stromal cells surrounding proximal airway cartilage in normal mouse lung is capable of enhancing proximal programs during lung morphogenesis.^737^ Up-to-date, only few FGF signaling-related gene therapies have entered clinical trials. NV1FGF is a plasmid-based angiogenic gene delivery system for local expression of FGF1. Intramuscular administration of NV1FGF resulted in a noteworthy reduced risk of major amputation in patients with critical limb ischemia.^738^ In 2017, phase II clinical trials for NV1FGF was completed and provided further support for its use in patients with severe peripheral artery occlusive disease.

The above-described molecules such as sFGFR2IIIc (S252W)^733,734^ or MEK inhibitor^739^ or glycosaminoglycans^740^ can partially alleviate the AS, but may bring undesired effects as they do not specifically antagonize the mutant FGFR2 itself. In contrast, RNA interference (RNAi) could inhibit the expression of mutant alleles at the transcriptional level. A short hairpin RNA (shRNA) targeting the dominant mutant form of *FGFR2* (*FGFR2* (S252W)) prevents the phenotypes of AS in mice.^739^ Safety and efficiency are the two major concerns for the application of RNAi-related therapeutics. AAV has unique advantages of gene transfer for therapeutic treatment of a number of diseases, including congenital blindness, hemophilia, and spinal muscular atrophy.^741,742^ Our group screened a siRNA specifically targeting the *FGFR2-P253R* allele, when this siRNA was delivered to the skulls in AS mouse model using AAV9 (AAV9-*FGFR2*-shRNA), it attenuated the premature closure of coronal suture and the decreased calvaria bone volume.^743^ Such biological strategy, in combination with other therapies including surgeries, provides experimental clues for the biological therapies of other genetic skeletal diseases.

In recent years, CRISPR/Cas9-based method has been is developed for gene therapy. Some studies have verified the advantage of CRISPR/Cas9 technology for the correction of human hereditary genetic diseases, such as liver diseases,^744^ cataract disorder,^745^ Duchenne muscular dystrophy,^746^ tyrosinemia,^747^ thalassemia,^748^ and so forth. Miao et al.^749^ found that Cas9 protein can achieve higher frequency of precise correction of the *FGFR3*-G374R mutation than Cas9 mRNA. These strategies completely suppressed phenotypes of ACH without off-target effects checked by whole-genome sequencing. CRISPR/Cas9 technology can precisely correct individual mutations with high fidelity and is potentially translatable for clinical therapies of human diseases, especially genetic diseases in the future.

## Conclusion and perspective

Knowledge of the role of FGF/FGFR signaling in pathological and physiological conditions has advanced considerably in the past decades. In this review, we summarized the structure and function of FGF signaling molecules and the detailed regulatory mechanisms. FGF/FGFR system contributes to the pathophysiology of multiple disorders in humans, including genetic diseases, dysplastic diseases, various types of cancer, metabolic disorders, and degenerative diseases, as well as injuries and regeneration. Much remains to be learned. The spatiotemporal expression patterns, accurate roles, and underlying mechanisms of individual FGFs/FGFRs in the development and diseases/injuries are largely unknown.

Activation of FGF signaling is tightly controlled with diverse transduction specificity, which mainly depends on the molecular structures of FGFs/FGFRs. With the advance of multiple disciplines including structure biology, we have acquired more information about FGFs/FGFRs, such as their structures, binding partners, key amino acids mediating the specific binding and signaling pathways. We need to know from the viewpoint of structure why individual FGFs have variable binding affinities of respective FGFRs; why the same FGF ligand bind distinct group of FGFRs at different concentration; the downstream signaling pathways activated by individual FGF through respective FGFR at different concentrations and in physiological and pathologic circumstance; can we switch the binding affinity of individual FGFs, based on their structure, to HS and FGFRs to have novel therapeutic effects on aberrant FGF signaling-related disease? With this information, we will have the possibility to fine tune FGF-related signaling to achieve better therapeutic outcome in the future.

There are complex interactions among individual FGFs and FGFRs. Most FGF can bind multiple FGFRs with differential binding affinities. So far, there are few studies about the differential signaling pathways activated by individual FGF through corresponding FGFRs. Considering the differential even opposite effects of each FGFR in the homeostasis maintenance and occurrence of diseases, for example, FGFR1 promotes while FGFR3 suppresses OA pathogenesis, the effects of individual FGF on OA and cartilage injuries are the summed effects of all signaling pathways of FGFRs activated by the applied FGF. More studies are needed to know the individual FGFRs activated by the applied FGFs at specific concentrations.

To obtain these knowledges, we need new strategies such as omics technology, single-cell analysis, and in vivo imaging, as well as utilization of more species of model animals and more spatiotemporally tunable genetic approaches. For example, our commonly used strategy to study the role of individual FGFs or FGFRs in the disease pathogenesis has limitation. We need to use conditional approach to spatiotemporally delete or overexpress individual FGFs or FGFRs in a certain type of cells, for example, chondrocytes, aimed to dissect the role of individual FGFs or FGFRs in the development and maintenance of the targeted cells. In addition, it is appreciated that mutations of individual FGFs or FGFRs can have detrimental effects, but a systematic understanding of intracellular pathway activation and dynamics is still lacking.^750^

To mimic the effects obtained from omics and conditional knockout study, we need to use targeted therapy approaches, which means to precisely modulate individual FGFs, FGFRs, and downstream signaling in specific types of cells at specific disease stages. The good news is that we are having more and more approach to exert these targeted treatments. For example, aptamer-based cell lineage or tissue targeting approaches are increasingly utilized. Several aptamers have been discovered to specifically target bone-forming site, osteoblasts, osteoclasts, and osteocytes in the skeletal tissue. We can similarly find aptamers specifically targeting for distinct cells at different growth phases, or inflammatory cells, paracancerous, and non-tumorous tissues, and so on.

FGF pathway interacts extensively with other signaling pathways during a variety of development and disease processes. Clarifying the interactions among FGF signaling, and these signaling pathways, such as BMP/TGF-β, PTH, hedgehog, and retinoid pathways, will provide us with the molecular bases for searching for combined therapies.^751^

Interventions targeting FGFs/FGFRs represent new approaches for the treatment of a wide range of diseases including genetic disorders, cancer, metabolic disease, degenerative disease, and injury repair. Developments in this field will likely be facilitated by structure-based drug design of agonists and antagonists for FGF signaling.
